# Predictive risk modeling for child maltreatment detection and enhanced decision-making: Evidence from Danish administrative data

**DOI:** 10.1371/journal.pone.0305974

**Published:** 2024-07-10

**Authors:** Michael Rosholm, Simon Tranberg Bodilsen, Bastien Michel, Albeck Søren Nielsen

**Affiliations:** 1 Department of Economics and Business Economics, Aarhus University, Aarhus, Denmark; 2 TrygFonden’s Centre for Child Research, Aarhus University, Aarhus, Denmark; 3 IZA Institute of Labor Economics, Bonn, Germany; 4 Centre for Integrated Register-based Research, Aarhus University, Aarhus, Denmark; 5 School of Economics and Management, Nantes University, Nantes, France; Università degli Studi di Torino: Universita degli Studi di Torino, ITALY

## Abstract

Child maltreatment is a widespread problem with significant costs for both victims and society. In this retrospective cohort study, we develop predictive risk models using Danish administrative data to predict removal decisions among referred children and assess the effectiveness of caseworkers in identifying children at risk of maltreatment. The study analyzes 195,639 referrals involving 102,309 children Danish Child Protection Services received from April 2016 to December 2017. We implement four machine learning models of increasing complexity, incorporating extensive background information on each child and their family. Our best-performing model exhibits robust predictive power, with an AUC-ROC score exceeding 87%, indicating its ability to consistently rank referred children based on their likelihood of being removed. Additionally, we find strong positive correlations between the model’s predictions and various adverse child outcomes, such as crime, physical and mental health issues, and school absenteeism. Furthermore, we demonstrate that predictive risk models can enhance caseworkers’ decision-making processes by reducing classification errors and identifying at-risk children at an earlier stage, enabling timely interventions and potentially improving outcomes for vulnerable children.

## Introduction

Child maltreatment is a widespread problem. Globally, up to one billion children have experienced physical, sexual, or psychological violence in the past year [1]. In particular, a quarter of all adults report having been physically abused during childhood, and one in five women and one in thirteen men report having been sexually abused as a child [2]. This type of abuse can have serious and lasting consequences on the physical and mental health of the victims throughout their lives [2, 3]. Consequentially, child maltreatment represents a significant strain on healthcare systems and major financial costs for most societies. In the US alone, the lifetime health-related burden associated with substantiated cases of child maltreatment incurred each year was estimated at USD 428 billion [4].

Child Protection Services (CPS) addresses child maltreatment by examining and investigating referrals of abuse and neglect reported by individuals or institutions. However, identifying which referrals require immediate attention and which children need to be removed from their homes is a challenging task with significant stakes. This task becomes even more demanding in the many countries that mandate CPS to promptly classify referrals based on their risk level upon receipt and to conduct thorough investigations into the most serious cases. Recent qualitative evidence from Denmark reveals significant differences in how Danish municipalities categorize and respond to referrals [5]. This inconsistency can lead to challenges in prioritizing and addressing cases appropriately. Due to the absence of a common categorization system, caseworkers frequently develop informal, individualized strategies to handle referrals. These pathways, based on professional judgment, aim to compensate for deficiencies in the formal system. While these informal methods can enhance responsiveness, they also introduce variability and potential inequities in how referrals are handled.

Predictive risk modeling uses past data to predict the likelihood of future adverse outcomes [6]. In the context of child welfare, a predictive risk model (PRM) has the potential to assist CPS in their efforts to identify cases of child maltreatment, by helping them better process the information they have at their disposal to assess a child’s risk and may help to reduce decision time and errors [6–8]. Additionally, it has the potential to increase the consistency with which caseworkers respond to similar cases, thereby reducing the extent of caseworkers’ biases and promoting fairness across different groups [7, 9].

Currently, caseworkers in Danish CPS do not utilize PRMs for decision-making in child welfare cases. Instead, they rely on various structured frameworks and methodologies designed to guide caseworkers in assessing risk and protective factors in child welfare cases [10]. It is worth noting that PRMs are already being utilized by CPS in some US counties to predict child maltreatment. For instance, CPS in Allegheny County, Pennsylvania, uses a PRM that leverages administrative data to compute risk scores. This model assists hotline workers in making decisions about whether phoned-in referrals should be screened-in for investigation [11]. Another model, the Eckerd Rapid Safety Feedback Process [12], is currently being used or tested in the states of Indiana, Florida, Maine, New Hampshire, Ohio, and Oklahoma.

In this study, we develop PRMs using Danish administrative data to investigate how accurately caseworkers’ most severe decision, the choice to remove a child from their family in response to a referral, reflects child maltreatment. We investigate the PRMs’ predictability of child maltreatment in two steps. First, we assess the extent to which the PRMs can predict child removal. Second, we conduct an extensive external validation study to determine whether the predictions from our preferred PRM are correlated with other adverse child outcomes.

Further, we explore whether these models have the potential to enhance CPS’ decision-making process. We do so in the context of the Danish welfare state, where caseworkers have a college education and should thus be well qualified to assess child maltreatment. A key motivation for the analysis is that maltreatment is not observed directly, either by caseworkers or in administrative data. For a PRM to have the potential to improve decision-making, it must therefore be trained to predict an outcome that is very closely related to maltreatment. If caseworkers make good predictions, then the decision to remove a child should be very closely related to (i) the information on which the removal decision is made; and (ii) other measures of child maltreatment.

Our study makes several contributions to the literature on algorithms for public policy purposes and to the literature on child maltreatment detection. First, we add to the limited body of evidence showing that administrative data in combination with machine learning methods can identify child maltreatment quite accurately [8, 13, 14]. We first establish the predictive properties of the PRM using inputs corresponding to the data that legally can be accessed by caseworkers in actual cases. Our best-performing PRM demonstrates good predictive capabilities, distinguishing between cases that result in removal and out-of-home placement, and those that do not, with a probability of 86%.

Second, we show that the predictive ability of the PRM does not improve much when we include a much larger set of information available to us as researchers, implying that caseworkers base their decisions to a large extent on sufficient information. Importantly, we also demonstrate that good predictive power can be achieved with a very limited set of explanatory variables, suggesting that access to extensive administrative data, such as Scandinavian data, is not a prerequisite for using PRMs in CPS.

Third, to support the notion that the model predicts not only child removal but also broader aspects of child maltreatment, we demonstrate its effectiveness through external validation. Our findings contribute to the existing literature by showing that predictions from a model designed to predict child removal also exhibit strong positive correlations with various adverse child outcomes across multiple domains, including crime, physical and mental health, and school absence. Despite these encouraging results, it is crucial to emphasize that we cannot claim the model directly predicts child maltreatment, as this remains inherently unobservable to us as researchers.

Fourth, we provide evidence that, if used appropriately, PRMs can potentially improve CPS decision-making process. Despite its importance, this study is, to our best knowledge, the first to provide evidence on this issue. To do this, we identify referrals for which CPS decided not to intervene during the mandatory four-month investigation period, but for which an intervention was carried out shortly after. We interpret these referrals as CPS errors (false negatives). In total, false negatives represent 15.2% of referrals for which no form of intervention was implemented by CPS during the investigation period. Strikingly, we find that more than 60% of these false negatives had a predicted maltreatment risk belonging to the highest 20% of the overall risk distribution.

Taken together, this paper provides the first combined evidence that (i) a PRM designed to predict removals offers a consistent ranking of referred children based on their risk of being removed from their homes and of experiencing a broad range of other child adversities; and (ii) this PRM has the potential to enhance decision-making in CPS, thereby improving the lives of vulnerable children.

## Background and institutional settings

Although Denmark is a generous welfare state with extensive and generous income transfers, free access to healthcare and education, low rates of poverty, and income inequality, child maltreatment remains a problem. In 2008 and 2009, 3.0% of children in Denmark suffered from physical neglect, 5.2% from emotional abuse, 5.4% from physical abuse, and 3.4% from sexual abuse [15]. These figures are lower than those reported in other parts of the world. Nevertheless, they should be considered with caution given the inherent difficulties associated with the measurement of child maltreatment and differences in institutional settings.

Alleged cases of child maltreatment are reported to Danish CPS using a standardized notification form, which can be submitted by any citizen. Public employees, institutions, and non-governmental organizations are required to notify CPS when there is a concern for a child’s well-being, while private citizens may also submit a referral of concern. In Table 1 we show the distribution of referral sources for referrals received by CPS from April 2014 to December 2018. Public authorities are the most important source of referrals. In 2018, schools were responsible for 22.5% of the referrals received by municipalities, health institutions for 17.2%, and the police and courts of justice for 9.6%.

**Table 1:** Number and sources of referrals by year.

	2014	2015	2016	2017	2018
Anonymous	0.060	0.053	0.062	0.063	0.066
Crisis centers or organizations	0.010	0.009	0.009	0.018	0.019
Day care	0.063	0.060	0.065	0.063	0.059
Family	0.093	0.089	0.089	0.085	0.085
Health institutions	0.159	0.161	0.165	0.171	0.172
Intergovernmental administration	0.062	0.069	0.062	0.107	0.118
Other	0.238	0.243	0.234	0.167	0.151
Place of placement	0.003	0.003	0.002	0.006	0.008
Police or court of justice	0.086	0.082	0.077	0.088	0.096
School	0.200	0.218	0.234	0.231	0.225
Unknown	0.026	0.013	0.000	0.000	0.000
Total number of notifications	64,756	93,064	100,487	113,204	120,870

*Notes:* This table shows the share of referral sources for each year between 2014 and 2018. In 2014, data spans only the period from April to December. The table disregards referrals concerning parents and children above the age of 18.

Immediate danger assessments are mandated within 24 hours, with removal required for high-risk cases. By law, further investigations must be conducted within four months to ascertain the necessity of an intervention. The mildest CPS intervention is to provide preventive services to the child and family, such as assigning a personal counselor, implementing support measures for parents, or sending the child to a boarding school. If CPS determines that these measures are insufficient, they may decide to remove the child from the home.

Recently, the number of referrals received by Danish CPS has grown substantially under increasing media attention. As displayed in Table 1, 93,064 referrals of child maltreatment were received in total in 2015 and 120,870 in 2018, a 29.9% increase. To put these figures into perspective, 1,160,550 children under 18 were residing in Denmark in 2018, meaning that the annual referrals per child ratio now exceeds one in ten. This development has increased the pressure on CPS, reducing the amount of time caseworkers can dedicate to each referral, which makes it even more important to look for new ways to assist them in identifying cases of maltreatment.

## Materials and methods

We employ a retrospective cohort study design, utilizing administrative data that includes information on all individuals residing in Denmark from April 2014 to December 2018. Importantly, these administrative registers include detailed individual-level information on (a) all referrals regarding child maltreatment received by CPS; (b) all preventive services implemented by CPS; and (c) all child removals.

As further described below, the administrative datasets also provide rich background information on each referred child and their family.

### Sample description

The PRMs were developed and evaluated using CPS referrals from April 2016 to December 2017. A total of 195,639 referrals (representing 102,309 children) were received during this period. A small subset was excluded due to unavailable data: (i) referrals for children still in their mother’s womb or above 17 years old at the time of referral (7,188 observations removed); and (ii) referrals for children not found in the registers within two years preceding the referral or emigrated less than four months after the referral (15,407 observations removed). This results in a sample of 173,044 referrals and 90,644 children.

Table 2 presents information on the 173,044 referrals included in our sample and provides a comparison with the full population of Danish children in 2017. The referred children are about ten years old on average, and less than half of the referrals involve girls. In 36.3% of cases, the children live with both parents, while in 39.7% of cases, they live with a single mother. This contrasts starkly with the full population of children in Denmark, where 72.5% live with both parents and 16.4% live with a single mother. The majority of referrals (66%) concern children who have been referred to CPS before. Additionally, 37% involve children who have already received preventive services, and 6.7% concern children who have been removed from their homes. Immigrants and their descendants are overrepresented in the referrals, accounting for 7.0% and 14.9%, respectively, compared to their shares in the overall population of Danish children, which are 3.8% and 9.1%, respectively.

**Table 2:** Sample description.

**Panel A: Children’s background information**			
Continuous variables	Mean	Std. dev.	Population mean
Age	9.846	4.760	9.249
Mother’s age at child birth	28.586	5.915	30.610
Father’s age at child birth	32.341	7.260	33.274
Number of moves	2.853	3.235	1.410
Number of referrals the previous 90 days	1.652	1.360	0.026
Number of referrals the previous 365 days	2.484	2.373	0.093
Number of referrals the previous 730 days	3.186	3.191	0.170
Number of preventive services the previous 730 days	0.470	0.930	0.131
Number of removals the previous 730 days	0.048	0.284	0.006
Categorical variables	%	N	Population %
		173,044	
Girl	46.1		48.7
Child living with both parents	36.3		72.5
Child living with mother in a new relationship	10.3		6.1
Child living with single mother	39.7		16.4
Child living with father in a new relationship	2.5		1.1
Child living with single father	7.0		2.6
Child not living with any of their parents	4.2		1.3
Immigrant	7.0		3.8
Descendant of immigrant(s)	14.9		9.1
Rest of population	78.1		87.0
Any past referral	66.0		14.5
Any past preventive service	37.0		6.7
Any past removal	6.7		1.0
**Panel B: CPS interventions**	%	N	Population %
		173,044	
Removal occurs within four months	3.6		0.1
Removal occurs within a year	6.9		0.2
Preventive service is received within four months	15.3		1.3
Preventive service is received within a year	26.5		2.9

*Notes:* In this table, we provide information on the referrals received by Danish municipalities between April 2016 and December 2017. These statistics are computed based on 173, 044 referrals, representing 90, 644 different children. The unit of observation in the first two columns is the referral. The population means in the last column are based on the entire population of children residing in Denmark as of December 31, 2017, corresponding to 1, 165, 496 children.

### Outcomes and information sets

#### Main outcome

The main problem with child maltreatment is that it is not directly quantifiable in the administrative registers. Hence, to implement a prediction model for child maltreatment, we have to proxy it by observable information. This is a standard problem in predictive risk modeling [16].

To proxy the occurrence of child maltreatment, we use a binary variable reflecting child removal within the mandatory four-month investigation period by CPS.

This choice of proxy is motivated by various factors. First, higher risks of child removal and placement are typically associated with extreme cases of maltreatment, which also indicate an increased likelihood of other adverse child outcomes. Table 3 corroborates the claim, presenting the prevalence of eight adverse outcomes among three groups of children in Denmark as of January 1, 2017: (i) not referred in 2017, (ii) referred in 2017 but not removed, and (iii) referred in 2017 and removed. The table reveals that referred children were more likely to experience adverse outcomes, and those removed were even more likely. For instance, only 0.4% of non-referred children were charged with an offense or a crime in 2017, compared to 6.1% of referred children and 15.3% of those who experienced removal. Thus, the proportion of children charged with an offense or a crime in 2017 was almost 40 times greater among those removed than among the non-referred population of children.

**Table 3:** Indicators of maltreatment in three distinct groups.

		(i)	(ii)	(iii)
(a)	Charged (0/1)	0.004	0.061	0.153
(b)	Victimized (0/1)	0.004	0.042	0.101
(c)	Somatic illness (0/1)	0.286	0.383	0.495
(d)	Fracture (0/1)	0.115	0.152	0.183
(e)	Mental illness (0/1)	0.015	0.119	0.225
(f)	Diagnosed with anxiety (0/1)	0.004	0.048	0.093
(g)	Fraction of damaged teeth	0.035	0.067	0.071
(h)	Fraction of school year with unauthorized school absence	0.008	0.039	0.052
Number of children		1,107,088	65,131	3,609

*Notes:* The table shows the average value of a set of adverse outcomes measured in 2017 for (i) the entire population of children residing in Denmark as of January 1, 2017, who were not involved in any referral in 2017; (ii) children involved in a referral in 2017 without a subsequent removal; and (iii) children involved in a referral in 2017 with a subsequent removal. For adverse outcome (g), the numbers are only based on 513,042, 29,456, and 1,469 observations respectively across the population groups. For adverse outcome (h), the number of observations across the three groups is 515,951, 36,568, and 1,633, respectively.

Second, child removals are infrequent but not extremely rare in Denmark. Table 2 illustrates that within the four months after a referral was received (Panel B), removal and subsequent placement occurred in 3.6% of the cases. This percentage increases to 6.9% when considering the 12 months following receipt of the referral. In contrast, child deaths, often used as an indicator of child maltreatment in the literature [8, 17], are exceedingly rare events in Denmark. For instance, only five children died due to homicide in 2019 [18].

Although other adverse events, as listed in Table 3, occur more frequently, they may not always have a direct link to maltreatment and may not be well-defined for children of all ages. Additionally, certain variables are not reported daily in the administrative registers. For example, school absence statistics are reported monthly, and somatic illnesses are reported yearly, posing challenges in determining whether a specific event occurred before or after a referral was received.

When using child removal as the outcome variable, it is important to consider the non-uniform age distribution of removals in Denmark. Most removals occur for teenagers or shortly after birth, which can introduce potential bias favoring these age groups in risk predictions. However, child maltreatment can occur across various age groups to a similar extent. To address this, we present analysis results with “age-neutralized” predictions in S1 Appendix. These results show that conclusions from the main analysis remain unchanged.

#### Alternative child maltreatment outcomes

In addition to using child removals as a proxy variable for child maltreatment, we also collect data on twenty other adverse child outcomes linked to child maltreatment to validate the model trained to predict child removals. The validation outcomes are selected to cover different aspects of child maltreatment to provide a nuanced view of the model’s performance. The complete description of twenty outcomes is presented in S1 Appendix. The outcomes related to crime, physical and mental health, dental quality, and school absence are all based on Danish administrative registers covering the full population of children over the period considered in this study. The outcomes related to well-being and personality traits are derived as proposed by Andersen *et al.* [19] using survey data from Danish public schools. Hence, measurement errors may influence these outcomes more than the register-based information, as they are not directly observable but rather estimated based on survey data.

The choice of adverse outcomes is also based on prior evidence from the literature that has established a relationship between child maltreatment and the development of mental disorders, fractures, criminal behavior, poor dental quality, and school absenteeism [20–24].

#### Information sets

We create two information sets. The first one (henceforth referred to as the “limited information set”) is based on the information available to caseworkers for case assessment. It includes details such as the municipality receiving the referral, date of receipt, notifier (e.g., school, health institution, family member, or police), type of concern registered, and whether the referral is considered severe (e.g., cases of violent or sexual abuse, parental drug abuse, abusive parenting, or inadequate care). This set also contains detailed data from CPS on past referrals, removals, and preventive services for the referred child’s municipality. Additionally, it provides information about parents and siblings, like their ages, number of siblings, living arrangements, household size, and household mobility history. For the full list of variables in the limited information set, please refer to S1 Appendix.

The second information set (henceforth referred to as the “full information set”) encompasses all the data included in the limited information set along with a broader range of additional information available in the administrative registers (see S1 Appendix for details). In addition to the limited information set, the full information set comprises data about the child’s criminal background, health, and school performance, as well as the parent’s education, labor market status, income, criminal background, and type of residence. It also contains a complete history of the child’s past interactions with all Danish CPS. We use the full information set to examine the importance of the information constraint faced by caseworkers, as not all information in this set is currently accessible to CPS caseworkers. Therefore, the potential use of PRMs by Danish CPS would be constrained to be based solely on the limited information set.

Since all variables in both information sets are derived from Danish administrative registers, the dataset has a low degree of missingness. In cases where data is missing, we impute the missing values using the median of the available data points in the training sample (as defined in the Methods section).

If a PRM were to be utilized by Danish CPS, the country’s legislation would mandate the exclusion of ethnicity and sex information of the child and parents from the model’s information set. Hence, we have excluded this type of information from both information sets, even though it is accessible to caseworkers. In unreported analyses, we find that including information on ethnicity and sex in the models does not significantly alter our results and conclusions.

### Methods

#### Predictive risk models

To predict child removal we estimate four different machine learning models with varying degrees of complexity.

To train and test these models, we randomly split the full sample of referrals into two samples: a training sample (70%) and a test sample (30%). The former comprises 120,395 referrals (representing 63,303 different children), while the latter contains 52,649 referrals (representing 27,341 different children). The level of randomization is the mother’s social security number. This allows us to ensure that referrals concerning children from the same mother are all either in one group or the other and hence to reduce arbitrary overfitting [13]. All four models are calibrated using the same sample splits and data so that the predictions made by the models are directly comparable.

The output of the models is the predicted probability that a child removal occurs within four months of the referral receipt. We translate these probabilities into risk scores as follows. First, we rank all predicted probabilities in the test set and divide them into ten equal groups, or deciles. Each decile represents 10% of the distribution, with the lowest decile containing the lowest predicted probabilities and the highest decile containing the highest. We then assign a risk score from 1 to 10 based on the decile in which each referral’s predicted probability falls. Referrals in the first decile receive a score of 1, indicating the lowest risk, while those in the tenth decile receive a score of 10, indicating the highest risk. This method ensures that the risk scores are evenly distributed and directly tied to the empirical probability distribution.

The first model we consider is the logistic regression model estimated using maximum likelihood. The model is widely used across various disciplines and is specifically designed for binary dependent variables. In our application, the model specifies the log odds of removal to be a linear function of the variables in the model. The model offers the advantage of easy interpretation as the estimated coefficients indicate the change in the log odds of the outcome per unit change in explanatory variables. The main drawback of the logistic regression model is that it assigns a non-zero weight to all included variables, which makes it more prone to overfit the data.

The second model is the logistic regression model with the Least Absolute Shrinkage and Selection Operator (LASSO) [25]. This model is a generalization of the standard logistic regression model as it additionally performs both variable selection and regularization to enhance the accuracy of its predictions. The amount of regularization in the logistic regression model is determined by the parameter λ. This parameter is selected using five-fold cross-validation, and we follow the recommendation of Krstajic *et al.* [26] and use the value of λ that maximizes the cross-validated AUC-ROC minus one standard error. To avoid overfitting problems, the five folds are chosen such that children from the same mother always belong to the same fold.

The third model is a tree-based random forest model developed by Breiman [27]. The random forest model uses bagging as an ensemble learning method, which involves training different decision trees on different random subsets of data in parallel. In addition to bagging, random forest models also perform a random selection of explanatory variables for each decision tree. The fact that the random forest model is based on decision trees, makes it suitable to detect nonlinearities in the relationship between the outcome and the explanatory variables. We implement the random forest algorithm with 1,000 trees, using the ranger R package [28]. We use a Bayesian optimization approach to find the optimal values for the number of variables the algorithm can use at each node (mtry) and for the minimal node size (min.node.size). More precisely, we search for the optimal hyperparameters that yield the highest AUC-ROC based on five-fold cross-validation on the training sample. The Bayesian optimization procedure is implemented using the mlrMBO R package [29].

The fourth predictive model is the extreme gradient boosting (XGBoost) model proposed by Chen & Guestrin [30]. XGBoost is a tree-based method that uses boosting as an ensemble learning method. Boosting entails training different individual models sequentially in a way that allows each new model to learn from the mistakes made by the previous models. XGBoost is a computationally efficient boosting model that can handle nonlinear features in data, such as interaction effects, in a data-driven way and works in the presence of missing data in the predictors. It also employs several techniques to prevent overfitting, including regularization, early stopping, and pruning. The model is estimated using the xgboost R package. To optimize the algorithm’s performance, we specify seven hyperparameters using Bayesian optimization. Using the terminology of the xgboost package, the hyperparameters we consider are the following: (i) max.depth; (ii) eta; (iii) gamma; (iv) subsample; (v) colsample_bytree; (vi) colsample_bynode; and (vii) min_child_weight. The remaining parameters are set to their default values. These hyperparameters are chosen to maximize the cross-validated area under the receiver operating characteristic curve across the five folds of data.

#### Predictive accuracy

The output of the models we consider is the predicted probability of the outcome variable. In this case, model performance is often assessed using the area under the receiver operating characteristic curve (AUC-ROC). The receiver operating characteristic (ROC) curve plots the true positive rate of a predictive model against its false positive rate for each decision threshold *τ* ∈ [0, 1]. Formally, the AUC-ROC gives the probability of a model assigning a higher predicted probability to a randomly chosen true positive (a child who is removed) than to a randomly chosen true negative. Hence, the AUC-ROC may be interpreted as the model’s ability to distinguish between referrals leading to child removal and those that do not. Note, that a model making predictions at random would lead to an AUC-ROC of 50%. The AUC-ROC is the main performance criterion in this paper.

We also consider the area under the precision-recall curve (AUC-PR) as a supplementary performance metric. In a context of highly imbalanced data, where the number of positives is considerably smaller than the number of negatives, it is particularly relevant to consider the AUC-PR criterion, which reflects a model’s ability to predict the positive class in the data [31].

The precision of a binary classifier is determined by the ratio of true positives to the total number of predicted positives, while recall corresponds to the true positive rate. By varying the classification threshold *τ* between zero and one for a specific prediction model, the precision-recall curve can be obtained, from which the AUC-PR can be calculated as the areal under this curve. An ideal model would achieve an AUC-PR value of one, while random guessing would yield a score equal to the proportion of positives in the data. Higher values of AUC-PR are associated with better model performance for a given dataset, although AUC-PRs should generally not be compared across different datasets.

## Results

In this section, we present the results of our analysis. The first part focuses on the effectiveness of the PRMs in predicting child removal. Using the best-performing model, we further examine whether the predictions are associated with other adverse child outcomes to establish the model’s external validity. The second part investigates whether PRMs can enhance CPS’s decision-making process.

### Predicting child removal

In Table 4, we report on the ability of the different models to predict child removal occurring in the four months after a referral was received. We study the performance of the models when they are estimated on the limited information set as well as on the full information set. In both instances, we present the AUC-ROCs obtained by each of the four models, the associated 95% confidence intervals (CI), and the AUC-PRs.

**Table 4:** Test sample performance of the predictive risk models.

	Limited information set				Full information set			
	AUC-ROC	95% CI		AUC-PR	AUC-ROC	95% CI		AUC-PR
**Panel A: All referrals**								
Logistic regression	83.93	83.06	84.79	17.26	84.77	83.95	85.60	16.97
Logistic regression w. LASSO	83.53	82.66	84.39	16.79	85.02	84.21	85.82	17.77
Random forest	85.69	84.87	86.52	20.05	86.83	86.08	87.58	21.79
XGBoost	85.91	85.09	86.73	21.18	87.34	86.63	88.05	23.20
Removal rate: 3.35%								
**Panel B: Referrals regarding children with no prior involvement with CPS**								
Logistic regression	82.20	78.01	86.40	7.90	83.76	79.66	87.87	8.07
Logistic regression w. LASSO	81.85	77.18	86.53	6.72	83.79	79.62	87.96	5.99
Random forest	84.98	80.70	89.26	21.12	87.47	83.57	91.38	25.65
XGBoost	85.91	81.79	90.02	22.28	90.26	87.29	93.23	25.59
Removal rate: 0.65%								
**Panel C: Referrals regarding children with previous involvement with CPS**								
Logistic regression	79.45	78.39	80.52	17.81	80.52	79.51	81.53	17.53
Logistic regression w. LASSO	78.78	77.71	79.86	17.42	80.96	79.97	81.95	18.46
Random forest	81.49	80.47	82.50	20.13	82.90	81.98	83.83	21.18
XGBoost	81.64	80.62	82.66	20.79	83.28	82.38	84.19	22.61
Removal rate: 4.73%								

*Notes:* The table provides AUC scores for the four models predicting child removal within four months. The models are calibrated on a training sample comprising 120,395 referrals (representing 63,303 unique children) and tested on a test sample comprising 52,649 referrals (representing 27,341 unique children) (Panel A). Results displayed in Panel B are calculated based on the 17,778 test sample referrals (representing 17,778 unique children) regarding children with no history with CPS. Results displayed in Panel C are calculated based on the 34,871 test sample referrals (representing 15,267 unique children) regarding children with a history with CPS. The estimation and evaluation period starts in April 2016 and ends in December 2017. The confidence intervals for AUC-ROC are calculated by bootstrap using test sample data.

Two conclusions can be drawn from the results. First, the overall predictive performance of the models using the full information set is good with AUC-ROCs ranging between 84% and 88% (Panel A). The AUC-PRs vary between 17% and 24%, which is considerable given that only 3.35% of the referrals result in child removal. The XGBoost model has the best performance followed by the random forest model under both AUC metrics. This pattern holds for both information sets as well as for most of the different subgroups we consider (see S1 Appendix for details). Although methodological differences exist between studies, the AUC-ROC scores obtained by our models are larger than reported in previous studies [8, 13].

Second, our analysis suggests that models with higher complexity and larger information sets only tend to perform slightly better than less complex models with smaller information sets. The observed performance gains from moving to more complex models and more extensive information sets are relatively modest, typically in the range of 1–2% in AUC scores (Panel A). However, using more complex models and more extensive information significantly increases the predictive performance for the subgroup of children with no prior involvement with CPS (Panel B). This suggests that these children pose the greatest information and processing constraints, which indicates that additional information beyond what is available to CPS is necessary for accurate predictions in this group. However, we do still obtain relatively high AUC scores for this subgroup with the limited information set in combination with the tree-based methods. This later finding is important because these are presumably the cases for which caseworkers have the least information available, making their decisions particularly challenging.

**Fig 1:**
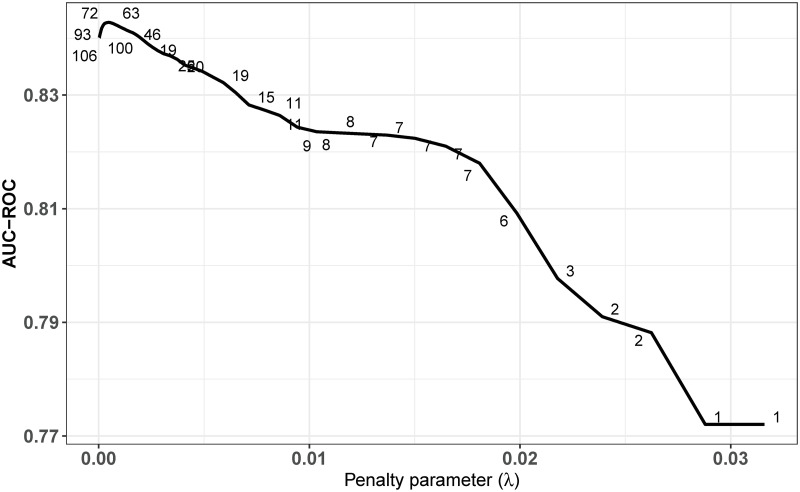
The effect of regularization on the predictive performance. The figure shows the AUC-ROC of the logistic regression model using LASSO feeding on the limited information set as a function of the penalty parameter. Higher values of the penalty parameter reduce the number of variables selected by the model. The AUC-ROC scores were calculated from the referrals included in the test sample. The numbers along the line indicate the number of variables selected by the model for certain values of the penalty parameter.

In Fig 1, we further analyze the importance of information by studying the extent to which prediction accuracy depends on the number of variables included in the model. To this end, we focus on the regularized logistic regression model and show how the AUC-ROC changes when the penalty parameter λ increases, which effectively reduces the number of variables included in the model. Interestingly, limiting the number of explanatory variables to seven only results in a reduction of the AUC-ROC by around two percentage points to 82%. The seven variables selected by this model are the following: (i) the number of referrals received in the past six months; (ii) the number of referrals received in the past two years; (iii) the number of preventive services implemented in the past year; (iv) the number of preventive services implemented in the past two years; (v) whether a preventive service was ongoing at the time the referral was received (0/1); (vi) the number of removals made in the past year; and (vii) whether a child lives with at least one of their parents (0/1).

These findings suggest that removal decisions can be predicted quite accurately based on a very limited set of information. This finding also indicates that the use of predictive risk modeling in child welfare settings may also be useful in countries where detailed administrative data is not available.

#### Identifying top predictors of the PRM

Next, we identify the most predictors in the XGBoost model using Shapley additive explanation (SHAP) values [32, 33]. SHAP values provide a model-agnostic approach to explain the contribution of each variable to the predictions of interest. They measure how much each variable influences the model’s predictions and can account for interactions and correlations among the variables. This enhances the interpretability and transparency of the model, which is essential for the practical usage of such models in child welfare settings.

**Fig 2:**
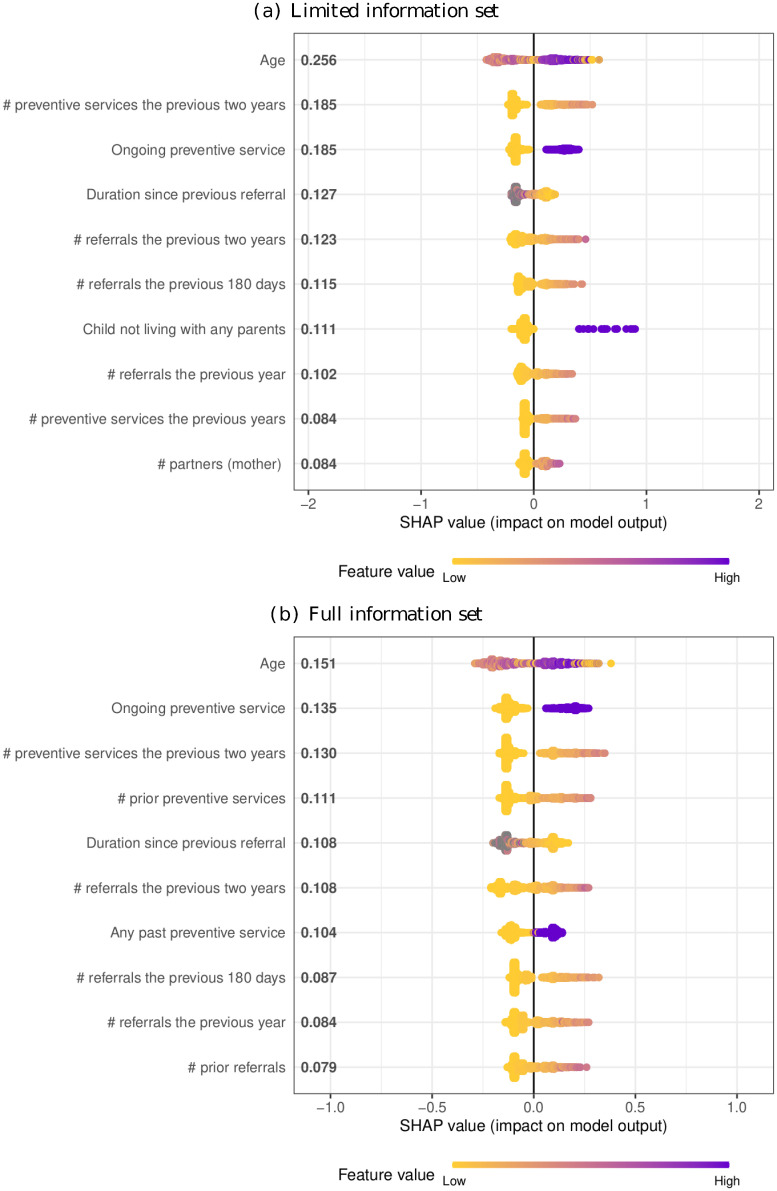
SHAP values for predicting child removals. This figure illustrates the SHAP values for the ten most influential variables based on the XGBoost model. The variables are ordered by descending variable importance as measured by the average absolute SHAP value. The figure is based on the referrals in the test set.

In Fig 2 we present the ten variables identified as being the most influential for predicting child removals in both the limited and the full model. In both cases, the age of the child is found to have the highest impact on the predictions, with a strongly heterogeneous effect on the predicted removal probabilities. The other identified variables by the SHAP methodology can broadly be categorized as variables related to prior or current involvement with CPS. This is also the case in the full model, where a much wider range of variables is included, suggesting that very detailed individual and family background variables are not a prerequisite for developing useful PRMs in a child welfare setting.

#### Internal validation

**Fig 3:**
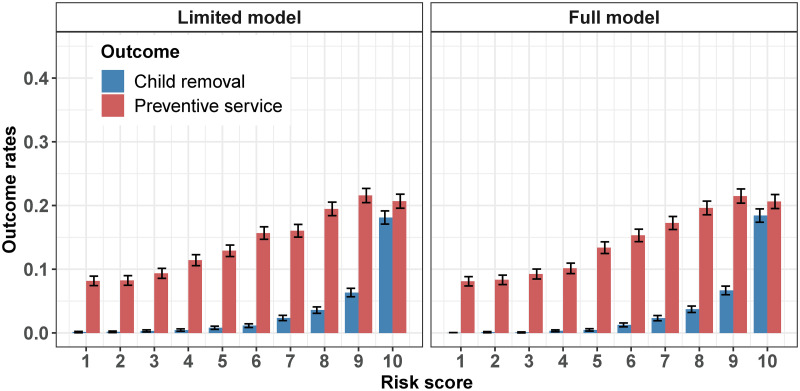
Observed intervention rates. The figure illustrates the observed rates of child removals and preventive services as a function of the risk scores generated by the XGBoost model, during the four-month period following the receipt of a referral. The figure is only based on test sample data. The vertical error bars correspond to the 95% confidence intervals.

In Fig 3, we display the observed four-month intervention rates among the individuals in our test sample within each decile of the predicted risk distribution generated by the XGBoost model. Reassuringly, we observe a monotonically increasing relationship between the risk score and the observed child removal rates. The relationship is convex, indicating that the XGBoost model effectively discriminates between severe and less severe cases. For instance, the removal rate is close to zero for referrals with a risk score of five or below but increases sharply to around 18% for referrals receiving a risk score of ten. We also observe a robust relationship between the risk score and the observed rates for preventive services, usually initiated before removal. However, we notice a higher likelihood of removal than preventive service initiation for referrals receiving a risk score of ten. This is indicative of the model’s ability to rank children based on their underlying risk of maltreatment consistently. Despite these promising results, it is essential to stress that we cannot assert the model directly predicts child maltreatment, as this phenomenon remains inherently unobservable to us as researchers.

### External validation

Next, we investigate whether our PRM is predictive of other child-adverse outcomes perceived to be associated with maltreatment beyond child removal.

To this end, we examine the relationship between the risk scores generated by the XGBoost model and a range of other adverse child outcomes which were all measured in 2018 after the last referral included in our sample was received. Similar analyses are presented in Vaithianathan *et al.* [34, 35], where they validate a PRM by considering outcomes from the health domain. In contrast, we consider a much wider range of outcomes from the domains of crime, physical and mental health, dental quality, well-being, and school absence. If the model is truly predictive of maltreatment, a necessary (though not sufficient) condition is that the risk scores generated by the model should be positively associated with the prevalence of these other adverse child outcomes.

**Fig 4:**
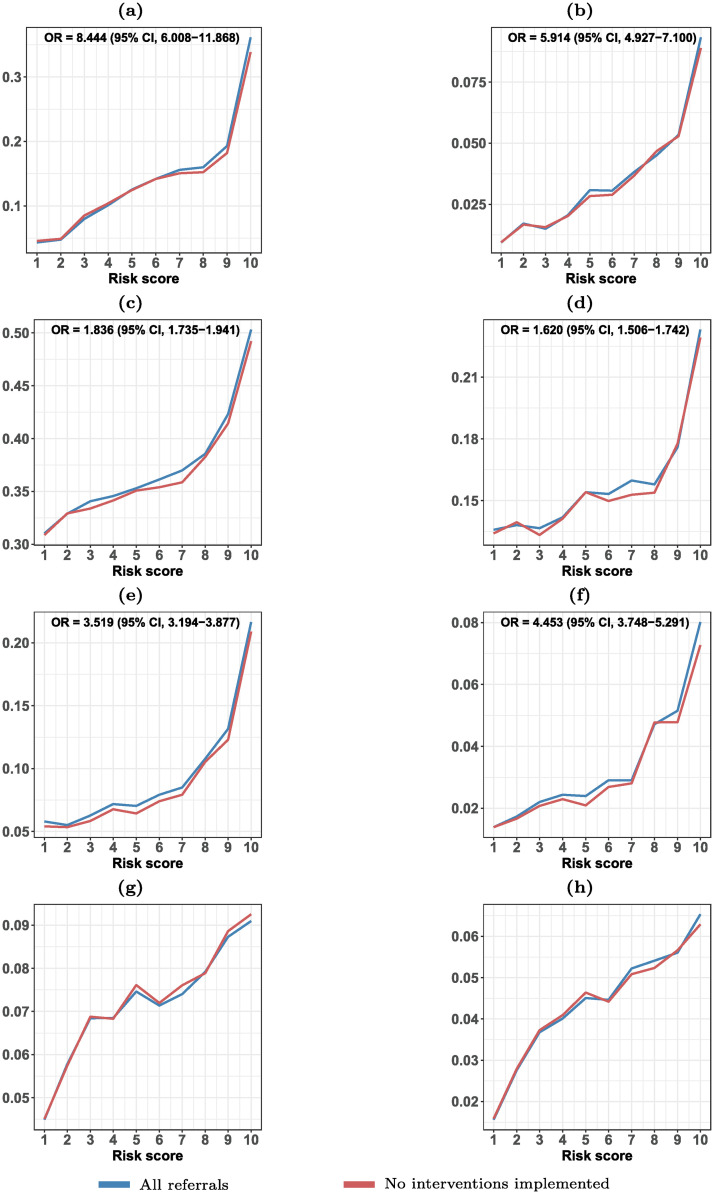
External validation. This figure shows the relationship between the predictions of the XGBoost models with the full information set and the risk of adverse child outcomes. Only the individuals in the test sample are used for this analysis. The blue line represents all referrals in the test sample, whereas the red line corresponds to the set of referrals in which CPS implemented no interventions in the first four months. The odds ratios for the binary outcomes compare the odds of the outcomes for risk scores 9 and 10 (high-risk cases) relative to the odds for risk scores 1 and 2 (low-risk cases) using all referrals. The numbers for charges are based only on children above the minimum age of criminal responsibility (15 years), while the numbers for school absence are based on children of compulsory school age (6–16 years). (a) Charged (0/1), (b) Victimized (0/1), (c) Somatic illness (0/1), (d) Fracture (0/1), (e) Mental illness (0/1), (f) Diagnosed with anxiety (0/1), (g) Fraction of damaged teeth, (h) Fraction of school year with unauthorized school absence. See S1 Appendix for further details about the outcomes.

The external validation results are presented in Fig 4, where the referrals are separated based on whether CPS decided to intervene within four months from the referral time. This helps us determine if cases where CPS chose not to intervene immediately have a similar risk of experiencing adverse outcomes as those where intervention was initiated when the risk score is taken into account.

The figure demonstrates a strong relationship between the risk scores and the prevalence of the eight outcomes (blue curves), indicating that models are predictive of child maltreatment more generally. For instance, our analysis shows that the odds ratio (OR) of being charged for an offense or a crime in 2018 is 8.44 (95% CI: 6.01–11.87) for high-risk referrals (risk scores of 9 or 10), compared to low-risk referrals (risk scores of 1 or 2). In the same vein, we find that the OR of being victimized in 2018 is 5.91 (95% CI: 4.93–7.10) for high-risk referrals relative to low-risk referrals. The figure also reveals that individuals with higher risk scores are prone to somatic and mental illnesses, experience poorer dental health, and have unauthorized absences from school.

Focusing on the referrals that did not result in any CPS intervention (red curves), we observe very similar results. We find that a higher predicted risk of removal is associated with a higher prevalence of each of the eight child-adverse outcomes in these cases. This finding rules out the possibility that the patterns discussed in the previous paragraph merely capture the disruptive impact of child removal on subsequent life. Moreover, it suggests that the model can detect high-risk cases beyond those where CPS decided to intervene. Interestingly, Fig 4 indicates the potential benefits of using PRMs to improve CPS decision-making. The figure shows that some high-risk children did not receive support from CPS, despite experiencing a high prevalence of adverse outcomes one year later. This suggests these children could have benefited from such support.

In S1 Appendix, we further demonstrate a strong positive relationship between the risk score and 12 additional adverse child outcomes, primarily related to mental health and well-being. This further reinforces the contention that the risk scores are indicative of a child’s likelihood of experiencing maltreatment in general, and not solely related to their likelihood of being removed from their home.

### Improving CPS decision-making process

In this section, we investigate the extent to which PRM has the potential to improve CPS decision-making process. We show that predictive risk models have the potential to help reduce errors made by CPS and speed up the implementation of appropriate measures to support at-risk children.

#### Reducing assessment errors by caseworkers

The effectiveness of PRMs in reducing errors made by CPS is a critical consideration. These errors can take the form of false positives, where CPS erroneously intervenes in a family, or false negatives, where CPS fail to intervene when it would have been the right decision to make. Despite its significance, there is currently no clear answer to this question in extant literature.

Occurrences of both false positives and false negatives are not straightforward to identify in the observed data. Nevertheless, we attempt to provide a crude estimate of the false negative rate by CPS by identifying referrals for which no intervention was implemented in the four months after the referral, but where an intervention was implemented in the following eight months. False positive cases, on the other hand, are more difficult to detect. For instance, a successful removal would often imply that the child who suffered from maltreatment can move back in with their parents after some time. Situations like this make it challenging to classify cases as false positives.

In Table 5, we use our definition of false negatives to investigate the extent to which our model would have flagged them as high-risk cases. To carry out this analysis, we focus on the 52,649 referrals in the test sample.

**Table 5:** Can predictive models help CPS identify cases of maltreatment?.

	All referrals			No intervention within four months		
	Share	Share of referrals with a risk score ≥ 9		Share	Share of referrals with a risk score ≥ 9	
		Limited information	Full information		Limited information	Full information
**Preventive service:**						
(a) Within 4 months	0.153	0.324	0.321			
(b) Between 4 and 8 months	0.075	0.289	0.284	0.087	0.270	0.265
(c) Between 8 and 12 months	0.036	0.268	0.277	0.042	0.235	0.244
**Child removal:**						
(d) Within 4 months	0.034	0.730	0.756			
(e) Between 4 and 8 months	0.020	0.612	0.653	0.018	0.601	0.640
(f) Between 8 and 12 months	0.017	0.578	0.591	0.014	0.601	0.612
(a) or (d)	0.177	0.377	0.380			
(b) or (e)	0.084	0.319	0.319	0.102	0.319	0.319
(c) or (f)	0.041	0.290	0.299	0.050	0.290	0.299

*Notes:* The table shows the share of referrals in the test sample for which CPS initiated an intervention in response to a referral. The risk scores referred to in this table are generated by the XGBoost model.

First, our estimation of CPS’s error rate suggests that there is room for improvement in caseworkers’ decision-making process. Indeed, we find that, in the four months following the end of the mandatory investigation period, a preventive service was implemented in 7.5% of the cases and a removal in 2% of the cases. Similar removal rates are found for 9 to 12 months after the receipt of a referral. This suggests that these results are not merely due to delays in the implementation of CPS decisions made during the mandatory investigation period, but rather to CPS’ decision not to intervene. Overall, 15.2% of the referrals identified as requiring no form of intervention were later reclassified as problematic.

Second, focusing on the cases that were reclassified after the end of the four months, we report their average decile risk score based on the XGBoost model at the time when the referral was received. Irrespective of the model specification, we find that a very significant share of these cases belongs to the very top of the risk distribution. Strikingly, more than 60% of the referrals for which a removal subsequently occurred receive a score of either 9 or 10, when the overall share of referrals with these risk scores by construction is 20%. Note that the models are trained to predict removal within the four months following receipt of a referral and not beyond. Consequently, there is no mechanical reason to expect that the models will necessarily predict removal accurately beyond these four months.

In S1 Appendix, we present descriptive statistics of the children for whom CPS made an error. This subgroup exhibits a particularly high prevalence of various negative child outcomes. Furthermore, when considering children with a risk score above 8, the observed rates are similar to those who experience removal within four months, indicating that the model correctly identifies the most vulnerable children at the top of the risk distribution.

#### Risk scores for removed children in past referrals

In this section, we examine whether the model can offer an early indication of children’s maltreatment risk, which is crucial for their well-being. We achieve this by analyzing the risk assessments provided by the model in the previous referrals leading up to the initiation of a CPS intervention. To do this, we focus on the children in the test sample who had at least five referrals from April 2016 to March 2017. We divide them into three exclusive subsets based on their status between April 2017 and December 2017: (i) children who experienced removal; (ii) children with preventive services initiated but no removal; and (iii) children without any intervention. For the last group, we randomly assign a placebo intervention date between April 2017 and December 2017.

**Fig 5:**
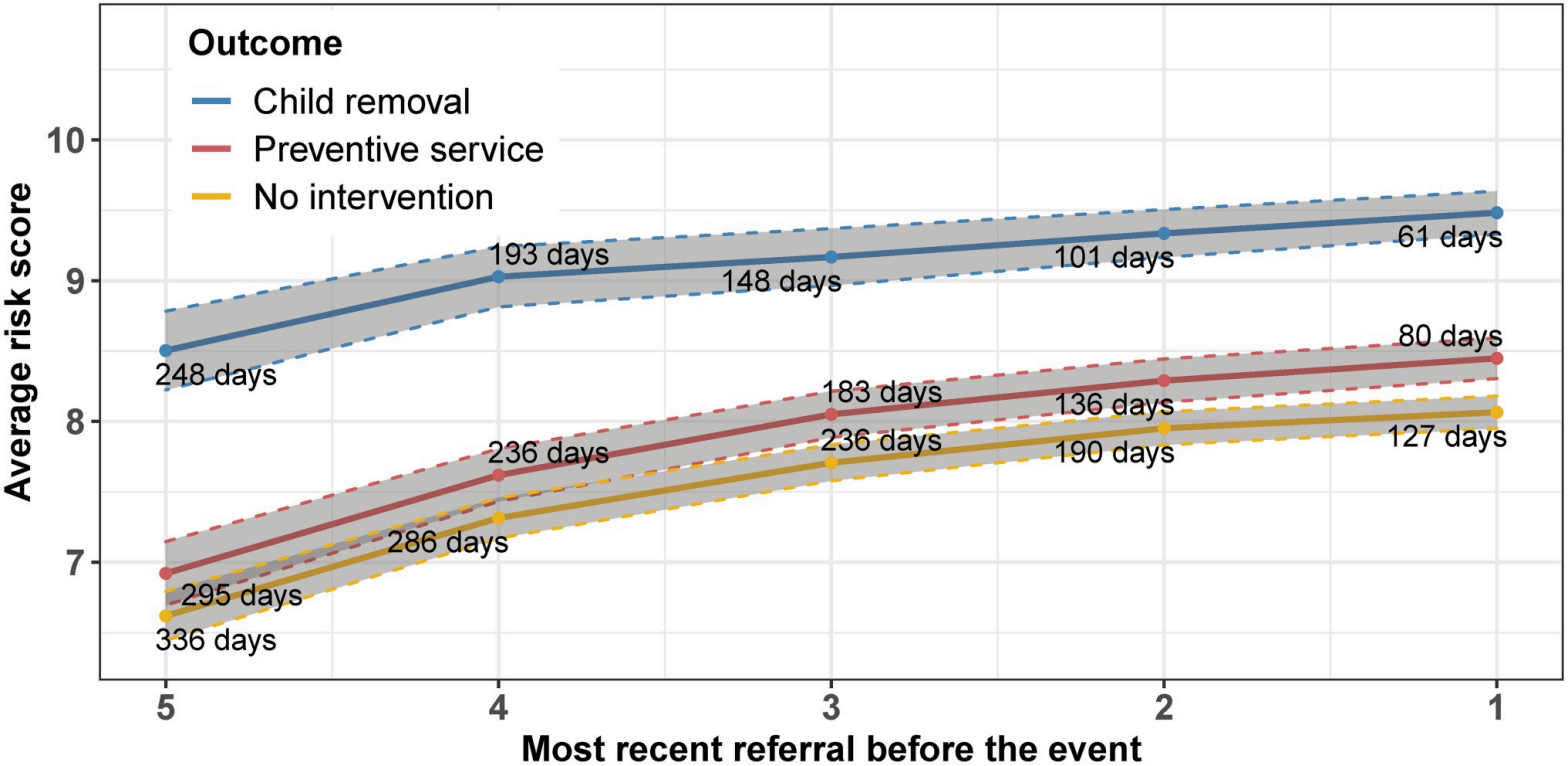
Risk score in past referrals. This figure depicts the average risk scores generated by the XGBoost model using full information for the most recent five referrals leading up to the initiation of a CPS intervention. The numbers displayed in the graph indicate the average number of days between the intervention date and the five most recent referrals.

Fig 5 presents the results of this analysis, showing the average decile risk scores for the five most recent referrals leading up to the event, along with the average duration in days between the event and the referral. Early referrals for children who are eventually removed have a significantly higher average risk score compared to the other two groups. The average risk score for removals in the earliest referral is approximately 8.5, 7 for preventive services and 6.6 for referrals without CPS intervention. As we move to the most recent referral, these risk scores increase to around 9.5, 8.4, and 8.1, respectively. Hence, the risk scores in earlier referrals are already higher for children where CPS eventually intervenes, especially for child removals, where the average risk scores are considerably elevated several months before the removal occurs.

Identifying cases of maltreatment is a challenging task, particularly in the context of limited resources. Hence, our results are promising, indicating that the use of PRMs may assist CPS in identifying cases of child maltreatment and accelerating the implementation of appropriate measures to support at-risk children.

## Discussion

In this study, we analyze how predictive risk modeling can serve to reach the public health goal of helping CPS detect child maltreatment. Using machine learning methods, we construct PRMs to predict child removals with the overarching aim of assessing the risk of child maltreatment among referred children. The best-performing PRM demonstrates good predictive power, as the probability of distinguishing between a removal case and a non-removal case exceeds 87%. Our findings indicate that PRMs can achieve good predictive power even with limited input variables, suggesting their potential usefulness in countries without comprehensive administrative data.

External validation of the PRM confirms its positive correlation with various adverse child outcomes related to maltreatment, indicating a consistent ranking of children by maltreatment risk. Additionally, we demonstrate that predictive risk modeling can aid in reducing CPS errors by identifying high-risk referrals that would have been overlooked otherwise and by accelerating the implementation of CPS intervention.

Extant literature suggests that there is room for improvement in caseworkers’ decision-making process [36, 37] This can be exemplified through the considerable inconsistency in decisions among child welfare experts and child protection caseworkers [38–44]. The use of PRMs in child protection settings has the potential to alleviate these issues by standardizing CPS responses to cases with similar characteristics, thus reducing the impact of individual caseworker beliefs and biases on case outcomes.

Rigorous evaluation of predictive risk modeling in child welfare settings is sparse. A recent example is the evaluation of the Eckerd Rapid Safety Feedback Process [12]. The evaluation shows that a PRM-based intervention was not able to reduce the incidence of children who experienced severe physical, sexual, or neglect maltreatment among children already known to a state child welfare agency. Another example is the evaluation of The Alleghany Family Screening Tool implemented in Alleghany County, Pennsylvania [45]. This study found that the tool resulted in a slight decrease in unnecessary child welfare screen-ins and a slight increase in cases that should have been screened-in for further investigation, along with evidence of reduced racial disparities.

We have not yet tested our proposed PRM on actual cases; hence, the current article provides only preliminary evidence regarding the potential of using predictive risk modeling in CPS. This limitation makes direct comparisons with the studies mentioned above challenging. Nevertheless, our analyses suggest that predictive risk modeling may help improve CPS’s decision-making process. However, several universally applicable conditions must be met to ensure its safe and effective use.

First, a general concern with PRMs is that they can perpetuate and even amplify inequalities between groups [46–48]. As mentioned in the Material and Methods section, we have omitted sensitive variables such as sex and ethnicity from the model. However, this does not rule out that the model is free from biases along these dimensions. For instance, if ethnic minorities historically have been excessively reported and investigated, the training data will reflect this. Even without direct input of ethnicity, variables like income level or family structure may correlate with ethnicity, potentially leading the model to associate certain socioeconomic conditions, which disproportionately affect ethnic minorities, with higher risk and prompting a more urgent investigation of these cases. Therefore, it is crucial to prospectively implement monitoring procedures to ensure that the use of the tool does not exacerbate inequalities between groups.

Second, the PRM must be regularly recalibrated to ensure that the cases it was calibrated on are similar to those it is being used for. During a transitory shock, such as the COVID-19 pandemic, which is likely to change the characteristics of the referrals received, the use of predictive models must be approached with great caution or discontinued altogether. It is also important to note that because caseworkers’ decisions directly influence our proxy for maltreatment, this may lead to an apparent increase in the predictive power of the model as it is recalibrated, without any guarantee of more accurate detection of maltreatment.

Third, several concerns arise regarding how such models would be used in practice by CPS. An important consideration is whether these models could unduly influence caseworkers’ decision-making processes. There is a risk that too much trust would be placed in the algorithm [49], causing caseworkers to overlook relevant information not captured by the algorithm, potentially worsening decisions on whether to intervene. Therefore, PRMs should not be used by caseworkers without appropriate training, emphasizing that PRMs cannot replace their decision-making process and should only be used as a secondary tool. Regular refresher courses would also be warranted.

Fourth, legal issues regarding data law (e.g., the General Data Protection Regulation 2016/679 in the EU) and administrative law need consideration if the models are to be used in real-life situations. Additionally, parental consent may be required for caseworkers to use predictive models, potentially limiting the application of PRMs to less risky families [50].

Overall, if these concerns can be satisfactorily addressed, our results suggest that, when used with appropriate caution, the joint use of machine learning and administrative data may help CPS identify children at risk of maltreatment and alleviate the burden on children and societies.

## Supporting information

S1 Appendix Supplementary data description and analysis results. This appendix contains a detailed description of the variables included in the model and of the variables used to validate the model. The appendix also contains additional empirical results, including subgroup results and further external validation analysis.
(PDF)
