# Supplementary material for: Predictive risk modeling for child maltreatment detection and enhanced decision-making: Evidence from Danish administrative data
Source: PLoS One. 2024 Jul 10;19(7):e0305974. doi: 10.1371/journal.pone.0305974 (PMC11236184; doi:10.1371/journal.pone.0305974)
Supplement: S1 Appendix — This appendix contains a detailed description of the variables included in the model and of the variables used to validate the model. The appendix also contains additional empirical results, including subgroup results and further external validation analysis. (PDF) [file pone.0305974.s001.pdf]

Supporting information for

Predictive risk modeling for child maltreatment detection and enhanced decision-making: Evidence from Danish administrative data

A Additional data descriptions

Information sets

Table A.1. Summary of explanatory variables in the limited information set

	Mean	Std. dev.
**CHILDREN'S BACKGROUND INFORMATION**		
Age	9.846	4.760
Siblings	2.151	1.688
Prior referrals		
Days elapsed since latest referral	195.147	247.415
# prior referrals	3.180	3.225
Any past referrals	0.623	0.485
# referrals the previous 30 days	1.343	0.898
# referrals the previous 90 days	1.602	1.295
# referrals the previous 180 days	1.884	1.647
# referrals the previous 365 days	2.322	2.179
# referrals the previous 730 days	2.889	2.854
# severe referrals the previous 30 days	0.566	0.816
# severe referrals the previous 90 days	0.675	1.002
# severe referrals the previous 180 days	0.792	1.186
# severe referrals the previous 365 days	0.969	1.455
# severe referrals the previous 730 days	1.194	1.771
Prior preventive services		
# prior preventive services	0.687	1.290
# preventive services the previous 365 days	0.259	0.632
# preventive services the previous 730 days	0.424	0.885
# prior severe preventive services	0.164	0.506
# severe preventive services the previous 365 days	0.048	0.230
# severe preventive services the previous 730 days	0.078	0.305
Any past preventive service	0.331	0.470
Any on-going preventive service	0.213	0.409
Any past severe preventive service	0.121	0.326
Any on-going severe preventive service	0.067	0.249
Prior removals		
# past removals	0.068	0.302
# removals the previous 365 days	0.022	0.157
# removals the previous 730 days	0.033	0.199
Any past forced removal	0.009	0.096

Continued on next page

Table A.1 – *Continued from previous page*

Variable name	Mean	Std. dev.
Any forced removal the previous 365 days	0.003	0.053
Any forced removal the previous 730 days	0.004	0.063
Any past removal	0.057	0.232
Residential information		
# moves	2.853	3.235
Moved to a new municip. within the past 60 days	0.046	0.217
Moved to a new municip. within the past 180 days	0.089	0.319
Moved to a new municip. within the past 365 days	0.142	0.428
Moved to a new municip. within the past 730 days	0.227	0.583
**CHARACTERISTICS OF THE REFERRALS**		
# causes for concern	1.348	0.663
Severe referral	0.408	0.491
Month		
January	0.054	0.225
February	0.047	0.212
March	0.059	0.235
April	0.085	0.279
May	0.100	0.301
June	0.111	0.314
July	0.056	0.230
August	0.081	0.273
September	0.104	0.306
October	0.098	0.298
November	0.114	0.317
December	0.091	0.288
Cause for concern		
Drug abuse by the child	0.019	0.137
Crime committed by the child	0.049	0.216
School problems	0.077	0.266
Other worrying behavior	0.273	0.445
Mental or physical disability of the child	0.047	0.211
Health conditions	0.002	0.041
Assault against the child	0.097	0.296
Other types of child neglect	0.089	0.285
Drug abuse by a parent	0.082	0.274

Continued on next page

Table A.1 – *Continued from previous page*

Variable name	Mean	Std. dev.
Crime committed by a parent	0.007	0.081
Other worrying behavior	0.003	0.053
Mental or physical disability	0.066	0.248
High level of conflicts or violence at home	0.164	0.370
Inadequate care from a parent	0.119	0.324
Homelessness	0.041	0.199
Other	0.195	0.396
Unknown	0.020	0.139
Notifier		
Unknown	0.000	0.008
Intergovernmental administration	0.088	0.283
School	0.241	0.427
Day care	0.068	0.251
Health authorities	0.148	0.356
Health care	0.018	0.131
Police/court	0.076	0.265
Family	0.089	0.284
Anonymous	0.066	0.249
Placement	0.005	0.068
Crisis center/organizations	0.015	0.121
Other	0.188	0.390
**CHARACTERISTICS OF THE HOUSEHOLDS**		
Household structure		
Child living with both parents	0.363	0.481
Child living with mother in a new relationship	0.103	0.305
Child living with single mother	0.397	0.489
Child living with father in a new relationship	0.025	0.155
Child living with single father	0.070	0.256
Child not living with any of the parents	0.042	0.201
# siblings	2.151	1.688
# people in the household	4.774	33.649
# children in the household	2.269	1.544
Prior referrals		
# past referrals in the household	2.725	6.420
# referrals in the household the previous 30 days	0.766	1.536

Continued on next page

Table A.1 – *Continued from previous page*

Variable name	Mean	Std. dev.
# referrals in the household the previous 180 days	1.230	2.474
# referrals in the household the previous 365 days	1.664	3.401
# referrals in the household the previous 730 days	2.297	4.912
# prior referrals per household member	0.558	1.025
# referrals per household member the previous 30 days	0.164	0.304
# referrals per household member the previous 180 days	0.262	0.498
# referrals per household member the previous 365 days	0.351	0.661
# referrals per household member the previous 730 days	0.477	0.879
Prior preventive services		
# prior preventive services in the household	0.808	4.942
# prior preventive services in the household the previous 30 days	0.027	0.229
# prior preventive services in the household the previous 180 days	0.123	0.589
# prior preventive services in the household the previous 365 days	0.224	0.916
# prior preventive services in the household the previous 730 days	0.396	1.466
# prior preventive services per household member	0.154	0.413
# prior preventive services per household member the previous 30 days	0.005	0.045
# prior preventive services per household member the previous 180 days	0.025	0.115
# prior preventive services per household member the previous 365 days	0.045	0.168
# prior preventive services per household member the previous 730 days	0.080	0.253
Prior removals		
# prior removals in the household	0.096	1.086
# removals in the household the previous 30 days	0.002	0.055
# removals in the household the previous 180 days	0.007	0.113
# removals in the household the previous 365 days	0.017	0.213
# removals in the household the previous 730 days	0.035	0.397
# prior removals per household member	0.012	0.087
# removals per household member the previous 30 days	0.000	0.013
# removals per household member the previous 180 days	0.001	0.021
# removals per household member the previous 365 days	0.003	0.030
# removals per household member the previous 730 days	0.004	0.044
**CHARACTERISTICS OF THE MOTHERS**		
Mother dead	0.008	0.090
Unknown mother	0.006	0.079
Civil status		
Unknown civil status	0.021	0.142

Continued on next page

Table A.1 – *Continued from previous page*

Variable name	Mean	Std. dev.
Divorced	0.245	0.430
Married	0.367	0.482
Other	0.010	0.099
Single	0.358	0.479
Age at child birth	28.583	5.891
# children	2.715	1.382
# partners	1.412	0.674
**CHARACTERISTICS OF THE FATHERS**		
Father dead	0.020	0.138
Unknown father	0.040	0.197
Civil status		
Unknown civil status	0.081	0.273
Divorced	0.217	0.412
Married	0.372	0.483
Other	0.005	0.070
Single	0.325	0.468
Age at child birth	32.311	7.098
# children	2.684	1.448
# partners	1.363	0.661

Notes: In this table, we provide information on the children who were the object of a referral for maltreatment received by Danish municipalities between April 2016 and December 2017. These statistics are computed based on 173,044 referrals, representing 90,644 different children. Missing values are replaced by the median of the non-missing observations in the training sample. The unit of observation is the referrals.

Table A.2. Summary of explanatory variables in the full information set

	Mean	Std. dev.
**CHILDREN'S BACKGROUND INFORMATION**		
Age	9.846	4.760
Siblings	2.151	1.688
Prior referrals		
Days elapsed since the latest referral	198.756	251.407
# prior referrals	3.583	3.644
Any past referrals	0.660	0.474
# referrals the previous 30 days	1.367	0.935
# referrals the previous 90 days	1.652	1.359
# referrals the previous 180 days	1.970	1.754
# referrals the previous 365 days	2.484	2.373
# referrals the previous 730 days	3.186	3.191
# severe referrals the previous 30 days	0.577	0.842
# severe referrals the previous 90 days	0.698	1.046
# severe referrals the previous 180 days	0.832	1.258
# severe referrals the previous 365 days	1.041	1.577
# severe referrals the previous 730 days	1.319	1.964
Prior preventive services		
# prior preventive services	0.796	1.394
# preventive services the previous 365 days	0.280	0.658
# preventive services the previous 730 days	0.470	0.930
# prior severe preventive services	0.194	0.562
# severe preventive services the previous 365 days	0.051	0.238
# severe preventive services the previous 730 days	0.085	0.317
Any past preventive services	0.369	0.483
Any on-going preventive service	0.225	0.418
Any past severe preventive services	0.137	0.344
Any on-going severe preventive service	0.069	0.254
Prior removals		
# past removals	0.080	0.328
# removals the previous 365 days	0.023	0.162
# removals the previous 730 days	0.035	0.207
Any past forced removal	0.011	0.104
Any forced removal the previous 365 days	0.003	0.054
Any forced removal the previous 730 days	0.004	0.065
Any past removal	0.066	0.248

Continued on next page

Table A.2 – *Continued from previous page*

	Mean	Std. dev.
Residential information		
# moves	2.853	3.235
Moved to a new municip. the previous 60 days	0.046	0.217
Moved to a new municip. the previous 180 days	0.089	0.319
Moved to a new municip. the previous 365 days	0.142	0.428
Moved to a new municip. the previous 730 days	0.227	0.583
School information		
Not going to school	0.297	0.457
# school changes	0.843	1.308
# school changes the previous 180 days	0.124	0.354
# school changes the previous 365 days	0.225	0.493
# school changes the previous 730 days	0.383	0.694
Illegal school absence	0.038	0.086
Child registered at day care center	0.457	0.498
Special needs education		
Receives special needs education	0.141	0.348
Special class	0.101	0.301
Mental problems	0.029	0.167
No special needs	0.942	0.234
Other special needs	0.029	0.169
Crime-related information		
# victimizations	0.148	0.561
# severe victimizations	0.062	0.406
# victimizations the previous 365 days	0.068	0.325
# victimizations the previous 730 days	0.093	0.391
# severe victimizations the previous 365 days	0.027	0.207
# severe victimizations the previous 730 days	0.038	0.252
Victim of a crime	0.107	0.309
Victim of severe crime	0.045	0.206
# charges	0.157	4.391
Charged	0.038	0.192
# charges (juvenile)	0.159	1.379
Charged (juvenile)	0.058	0.235
Any charges	0.083	0.275
Jailed	0.013	0.115
# convictions	0.022	0.219

Continued on next page

Table A.2 – *Continued from previous page*

	Mean	Std. dev.
# severe convictions	0.004	0.077
Conviction	0.015	0.120
Severe conviction	0.004	0.062
Health information		
# GP visits	64.973	47.032
# GP visits the previous 30 days	0.682	1.539
# GP visits the previous 180 days	3.543	4.775
# GP visits the previous 365 days	6.814	7.838
# GP visits the previous 730 days	12.854	12.801
Somatic diagnoses		
# diagnoses with ICD-10 code: A00-E90	0.778	3.102
# diagnoses with ICD-10 code: G00-N99	2.884	6.399
# diagnoses with ICD-10 code: O00-P96	0.959	2.848
# diagnoses with ICD-10 code: Q00-Q99	0.504	3.163
# diagnoses with ICD-10 code: R00-R99	1.021	2.286
# diagnoses with ICD-10 code: S00-Y09	4.914	4.628
# diagnoses with ICD-10 code: Z00-Z99	4.709	4.564
# diagnoses with ICD-10 code: U00-U99	1.908	0.853
# somatic diagnoses	17.873	17.051
Mental diagnoses		
# psychologist sessions	0.491	1.525
# psychologist sessions the previous 30 days	0.053	0.290
# psychologist sessions the previous 180 days	0.139	0.604
# psychologist sessions the previous 365 days	0.204	0.830
# psychologist sessions the previous 730 days	0.291	1.112
# mental diagnoses	0.706	1.788
# diagnoses with ICD-10 code: F00-F09	0.000	0.023
# diagnoses with ICD-10 code: F10-F19	0.009	0.152
# diagnoses with ICD-10 code: F20-F29	0.026	0.486
# diagnoses with ICD-10 code: F30-F39	0.046	0.481
# diagnoses with ICD-10 code: F40-F48	0.163	0.829
# diagnoses with ICD-10 code: F50-F59	0.028	0.443
# diagnoses with ICD-10 code: F60-F69	0.010	0.198
# diagnoses with ICD-10 code: F70-F79	0.029	0.360
# diagnoses with ICD-10 code: F80-F89	0.183	0.913
# diagnoses with ICD-10 code: F90-F98	0.402	1.601

Continued on next page

Table A.2 – *Continued from previous page*

	Mean	Std. dev.
# diagnoses with ICD-10 code: F99	0.013	0.156
Mental diagnosis	0.188	0.391
Birth information		
Log birth weight	8.110	0.192
Log birth height	3.934	0.062
Dental information		
No caries	0.449	0.497
Occlusal caries	0.103	0.304
Approximal caries	0.161	0.367
Smooth surface caries	0.127	0.333
Unknown dental status	0.160	0.366
# teeth inured due to acute mechanical trauma	0.109	0.485
# teeth	24.215	2.922
# tooth surfaces	109.259	14.440
# teeth with gingivitis	0.773	2.487
# dental visits	4.218	3.405
# non-damaged teeth	22.691	3.675
Fraction of non-damaged teeth	0.943	0.103
**CHARACTERISTICS OF THE REFERRALS**		
# Causes for concern	1.348	0.663
Severe referral	0.408	0.491
Referral municip. different from residence municip.	0.088	0.283
Month		
January	0.054	0.225
February	0.047	0.212
March	0.059	0.235
April	0.085	0.279
May	0.100	0.301
June	0.111	0.314
July	0.056	0.230
August	0.081	0.273
September	0.104	0.306
October	0.098	0.298
November	0.114	0.317
December	0.091	0.288

Continued on next page

Table A.2 – *Continued from previous page*

	Mean	Std. dev.
Cause for concern		
Drug abuse by the child	0.019	0.137
Crime committed by the child	0.049	0.216
School problems	0.077	0.266
Other worrying behavior	0.273	0.445
Mental or physical disability of the child	0.047	0.211
Health conditions	0.002	0.041
Assault against the child	0.097	0.296
Other types of child neglect	0.089	0.285
Drug abuse by a parent	0.082	0.274
Crime committed by a parent	0.007	0.081
Other worrying behavior	0.003	0.053
Mental or physical disability	0.066	0.248
High level of conflicts or violence at home	0.164	0.370
Inadequate care from a parent	0.119	0.324
Homelessness	0.041	0.199
Other	0.195	0.396
Unknown	0.020	0.139
Notifier		
Unknown	0.000	0.008
Intergovernmental administration	0.088	0.283
School	0.241	0.427
Day care	0.068	0.251
Health authorities	0.148	0.356
Health care	0.018	0.131
Police/court	0.076	0.265
Family	0.089	0.284
Anonymous	0.066	0.249
Placement	0.005	0.068
Crisis center/organizations	0.015	0.121
Other	0.188	0.390
**CHARACTERISTICS OF THE HOUSEHOLDS**		
Region of residence		
Capital	0.288	0.453
Zealand	0.163	0.369

Continued on next page

Table A.2 – *Continued from previous page*

	Mean	Std. dev.
Southern Denmark	0.216	0.411
Central Denmark	0.197	0.398
Northern Denmark	0.136	0.343
Household structure		
Child living with both parents	0.363	0.481
Child living with mother in a new relationship	0.103	0.305
Child living with single mother	0.397	0.489
Child living with father in a new relationship	0.025	0.155
Child living with single father	0.070	0.256
Child not living with any of their parents	0.042	0.201
# siblings	2.151	1.688
# people in the household	4.774	33.649
# children in the household	2.269	1.544
Socioeconomic status for the household member with the highest income		
In employment	0.493	0.500
Not in employment	0.414	0.493
Self employed	0.025	0.157
Other	0.068	0.251
Family disposable income	333,417.218	249,646.091
Accommodation type		
Owner-occupied home	0.245	0.430
Rented housing	0.755	0.430
Homeless	0.005	0.073
Farmhouse	0.042	0.200
Single-family house	0.344	0.475
Townhouse	0.158	0.365
Apartment	0.432	0.495
Dormitory	0.001	0.032
Other type of housing	0.009	0.096
Multifamily housing	0.009	0.096
Prior referrals		
# past referrals in the household	3.462	8.697
# referrals in the household the previous 30 days	0.794	1.590
# referrals in the household the previous 180 days	1.355	2.751
# referrals in the household the previous 365 days	1.917	4.071
# referrals in the household the previous 730 days	2.805	6.331

Continued on next page

Table A.2 – *Continued from previous page*

	Mean	Std. dev.
# prior referrals per household member	0.674	1.222
# referrals per household member the previous 30 days	0.169	0.314
# referrals per household member the previous 180 days	0.283	0.539
# referrals per household member the previous 365 days	0.394	0.749
# referrals per household member the previous 730 days	0.560	1.034
Prior preventive services		
# prior preventive services in the household	1.215	7.035
# prior preventive services in the household the previous 30 days	0.031	0.243
# prior preventive services in the household the previous 180 days	0.149	0.670
# prior preventive services in the household the previous 365 days	0.278	1.089
# prior preventive services in the household the previous 730 days	0.513	1.881
# prior preventive services per household member	0.211	0.501
# prior preventive services per household member the previous 30 days	0.006	0.047
# prior preventive services per household member the previous 180 days	0.029	0.121
# prior preventive services per household member the previous 365 days	0.053	0.181
# prior preventive services per household member the previous 730 days	0.095	0.279
Prior removals		
# prior removals in the household	0.234	2.114
# removals in the household the previous 30 days	0.003	0.063
# removals in the household the previous 180 days	0.013	0.171
# removals in the household the previous 365 days	0.033	0.350
# removals in the household the previous 730 days	0.076	0.731
# prior removals per household member	0.025	0.140
# removals per household member the previous 30 days	0.001	0.013
# removals per household member the previous 180 days	0.002	0.024
# removals per household member the previous 365 days	0.004	0.037
# removals per household member the previous 730 days	0.008	0.060
**CHARACTERISTICS OF THE MOTHERS**		
Mother dead	0.008	0.090
Unknown mother	0.006	0.079
Education		
Unknown level of education	0.020	0.139
Primary school	0.411	0.492
High school	0.063	0.243
Vocational	0.279	0.449

Continued on next page

Table A.2 – *Continued from previous page*

	Mean	Std. dev.
Higher education	0.025	0.156
Bachelor's degree	0.116	0.320
Master's degree/PhD	0.042	0.200
Missing	0.044	0.206
Civil status		
Unknown civil status	0.021	0.142
Divorced	0.245	0.430
Married	0.367	0.482
Other	0.010	0.099
Single	0.358	0.479
Age at child birth	28.583	5.891
# children	2.715	1.382
# partners	1.412	0.674
Labor market		
Disposable income (1000 DKK)	202.166	90.140
In employment	0.365	0.481
Social security recipient	0.174	0.379
Crime-related information		
# charges	1.924	8.065
Charged	0.428	0.495
Jailed	0.114	0.317
**CHARACTERISTICS OF THE FATHERS**		
Father dead	0.020	0.138
Unknown father	0.040	0.197
Education		
Unknown level of education	0.012	0.110
Primary school	0.379	0.485
High school	0.046	0.210
Vocational	0.326	0.469
Higher education	0.039	0.193
Bachelor's degree	0.057	0.232
Master's degree/PhD	0.049	0.216
Missing	0.091	0.288
Civil status		
Unknown civil status	0.081	0.273

Continued on next page

Table A.2 – *Continued from previous page*

	Mean	Std. dev.
Divorced	0.217	0.412
Married	0.372	0.483
Other	0.005	0.070
Single	0.325	0.468
Age at child birth	32.311	7.098
# children	2.684	1.448
# partners	1.363	0.661
Labor market		
Disposable income (1000 DKK)	212.114	213.839
In employment	0.537	0.499
Social security recipient	0.140	0.347
Crime-related information		
# charges	11.705	29.901
Charged	0.790	0.407
Jailed	0.398	0.490

Notes: In this table, we provide information on the children who were the object of a referral for maltreatment received by Danish municipalities between April 2016 and December 2017. These statistics are computed based on 173,044 referrals, representing 90,644 different children. Missing values are replaced by the median of the non-missing observations in the training sample. The unit of observation is the referrals.

Validation data

Table A.3. Description of data in the external validation

Variable name	Description	Source	Time span
**Main outcomes**			
Charged	This variable indicates whether the child has been charged for a crime. Only available for children over the criminal age of responsibility (15 years)	KRSI delivered by Statistics Denmark	1980–2018
Victimized	This variable indicates whether the child has been a victim of a criminal offense. Available for all children	KROF delivered by Statistics Denmark	2001–2018
Somatic illness	This variable indicates whether the child has been assigned any somatic diagnosis according to the ICD-10 classification. Available for all children	LPR_DIAG delivered by Statistics Denmark	1977–2018
Fracture	This variable indicates whether the child has been assigned any diagnosis in the "S" category using the ICD-10 classification. Available for all children	LPR_DIAG delivered by Statistics Denmark	1977–2018
Mental illness	This variable indicates whether the child has been assigned any diagnosis in the "F" category using the ICD-10 classification. Available for all children	PSYK_DIAG delivered by Statistics Denmark	1977–2018
Diagnosed with anxiety	This variable indicates whether the child has been assigned any diagnosis in the range F40–F43 using the ICD-10 classification	PSYK_DIAG delivered by Statistics Denmark. Available for all children	1977–2018
Fraction of damaged teeth	This variable measures the number of teeth with a non-empty DMFT index relative to the total number of teeth. Available for children in public schools	SCOR delivered by The Danish Health Data Authority	1995–2018

Continued on next page

Table A.3 – *Continued from previous page*

Variable name	Description	Source	Time span
Fraction of school year with unauthorized school absence	This variable measures the number of days with unauthorized absence relative to the total number of days in the school year. Unauthorized absence refers to any absence not due to illness, medical or dental appointments, or disability, and without the approval of the school administration. Available for children in public schools	FRAVAER delivered by "Styrelsen for It og Læring"	2011–2019
**Additional outcomes**			
Self-harm	This variable indicates whether the child has been assigned any diagnosis in the "X" or "Y" categories using the ICD-10 classification. Available for all children	LPR_DIAG delivered by Statistics Denmark	1977–2018
ADHD	This variable indicates whether the child has been assigned any diagnosis with ICD-10 code F90	PSYK_DIAG delivered by Statistics Denmark. Available for all children	1977–2018
Conduct disorder	This variable indicates whether the child has been assigned any diagnosis with ICD-10 code F91	PSYK_DIAG delivered by Statistics Denmark. Available for all children	1977–2018
Personality disorder	This variable indicates whether the child has been assigned any diagnosis with ICD-10 code F60	PSYK_DIAG delivered by Statistics Denmark. Available for all children	1977–2018
Depression	This variable indicates whether the child has been assigned any diagnosis in the range F32–F39 using the ICD-10 classification. Available for all children	PSYK_DIAG delivered by Statistics Denmark	1977–2018
Eating disorder	This variable indicates whether the child has been assigned any diagnosis with ICD-10 code F50	PSYK_DIAG delivered by Statistics Denmark. Available for all children	1977–2018

Continued on next page

Table A.3 – *Continued from previous page*

Variable name	Description	Source	Time span
Social well-being	This variable is a measure of social well-being proposed by [19] using the Danish well-being survey. Available for children in grades 4-9 in public schools. The variable has been standardized (z-score) at the population level	TRIVSEL delivered by "Styrelsen for It og Læring"	2015–2018
Neuroticism	This variable is a measure of neuroticism proposed by [19] using the Danish well-being survey. Available for children in grades 4-9 in public schools. The variable has been standardized (z-score) at the population level	TRIVSEL delivered by "Styrelsen for It og Læring"	2015–2018
Agreeableness	This variable is a measure of agreeableness proposed by [19] using the Danish well-being survey. Available for children in grades 4-9 in public schools. The variable has been standardized (z-score) at the population level	TRIVSEL delivered by "Styrelsen for It og Læring"	2015–2018
Conscientiousness	This variable is a measure of conscientiousness proposed by [19] using the Danish well-being survey. Available for children in grades 4-9 in public schools. The variable has been standardized (z-score) at the population level	TRIVSEL delivered by "Styrelsen for It og Læring"	2015–2018
Academic self-perception	This variable is a measure of academic self-perception proposed by [19] using the Danish well-being survey. Available for children in grades 4-9 in public schools. The variable has been standardized (z-score) at the population level	TRIVSEL delivered by "Styrelsen for It og Læring"	2015–2018
Fraction of teeth with gingivitis	This variable measures the number of teeth with gingivitis relative to the total number of teeth. Available for children in public schools	SCOR delivered by The Danish Health Data Authority	1995–2018

B AUCs for different population groups

In this section, we provide summaries of the predictive performance of the models for different subsets of the full set of referrals. Table B.1 shows the performance of the models for boys and girls. In Table B.2, we show the results for children of non-Western and Western origin, respectively. Table B.3 shows the predictive performance of the models for children of a low- and high socioeconomic status (SES). Finally, Table B.4 presents the results by age groups.

Table B.1. Results for boys and girls

Method	Limited set of covariates				Full set of covariates			
	AUC-ROC	95% CI		AUC-PR	AUC-ROC	95% CI		AUC-PR
**Panel A: Boys**								
Logistic regression	84.45	83.23	85.66	18.06	85.46	84.37	86.54	17.99
Logistic regression w. LASSO	85.07	83.92	86.22	18.88	86.11	85.05	87.17	19.18
Random forest	86.84	85.72	87.96	23.61	87.85	86.84	88.87	24.21
XGBoost	86.69	85.55	87.82	23.64	87.94	86.97	88.90	25.51
Removal rate: 3.30%								
**Panel B: Girls**								
Logistic regression	83.32	82.08	84.56	16.37	83.98	82.73	85.23	16.06
Logistic regression w. LASSO	81.76	80.46	83.05	14.53	83.75	82.52	84.98	16.45
Random forest	84.35	83.13	85.57	17.06	85.63	84.51	86.75	19.50
XGBoost	84.99	83.81	86.17	18.98	86.64	85.59	87.69	21.05
Removal rate: 3.42%								

Notes: The table provides AUC scores and the associated 95% confidence interval for the four predictive models. The AUC scores for boys are based on 28,527 referrals (representing 14,904 unique children) from the test sample. The AUC scores for girls are based on 24,122 referrals (representing 12,437 unique children) from the test sample. The estimation and evaluation period starts in April 2016 and ends in December 2017. The confidence intervals for AUC-ROC are calculated by bootstrap using test sample data.

Table B.2. Results for children of non-Western and Western origin

Method	Limited set of covariates				Full set of covariates			
	AUC-ROC	95% CI		AUC-PR	AUC-ROC	95% CI		AUC-PR
**Panel A: Children of non-Western origin**								
Logistic regression	89.00	87.41	90.58	22.49	87.91	86.12	89.70	20.15
Logistic regression w. LASSO	87.04	85.15	88.94	21.97	87.66	85.77	89.55	23.48
Random forest	90.54	88.95	92.13	27.47	90.79	89.26	92.33	26.42
XGBoost	90.97	89.47	92.47	30.89	91.22	89.79	92.65	29.57
Removal rate: 3.03%								
**Panel B: Children of Western origin**								
Logistic regression	82.76	81.76	83.76	16.37	84.10	83.18	85.02	16.49
Logistic regression w. LASSO	82.70	81.73	83.67	15.75	84.39	83.49	85.28	16.82
Random forest	84.61	83.67	85.54	18.64	85.92	85.07	86.77	20.90
XGBoost	84.75	83.82	85.69	19.55	86.48	85.68	87.28	22.08
Removal rate: 3.43%								

Notes: The table provides AUC scores and the associated 95% confidence interval for the four predictive models. The AUC scores for children of Western origin are based on 42,709 referrals (representing 22,017 unique children) from the test sample. The AUC scores for children of non-Western origin are based on 9,940 referrals (representing 5,324 unique children) from the test sample. The estimation and evaluation period starts in April 2016 and ends in December 2017. The confidence intervals for AUC-ROC are calculated by bootstrap using test sample data.

Table B.3. Results for children of low and high SES

Method	Limited set of covariates				Full set of covariates			
	AUC-ROC	95% CI		AUC-PR	AUC-ROC	95% CI		AUC-PR
**Panel A: Low-SES children**								
Logistic regression	83.54	82.60	84.49	18.36	84.46	83.57	85.35	17.45
Logistic regression w. LASSO	83.10	82.15	84.04	17.98	84.53	83.64	85.42	18.63
Random forest	85.28	84.37	86.19	21.77	86.31	85.47	87.15	22.85
XGBoost	85.64	84.74	86.54	22.52	86.98	86.19	87.77	24.49
Removal rate: 3.62%								
**Panel B: High-SES children**								
Logistic regression	84.94	82.60	87.29	10.55	85.37	83.09	87.64	13.45
Logistic regression w. LASSO	84.94	82.76	87.13	9.78	86.92	85.01	88.83	11.80
Random forest	87.28	85.31	89.24	11.33	89.13	87.51	90.74	14.50
XGBoost	86.65	84.57	88.72	11.93	88.81	87.16	90.45	13.59
Removal rate: 2.12%								

Notes: The table provides AUC scores and the associated 95% confidence interval for the four predictive models. The AUC scores for low-SES children are based on 43,187 referrals (representing 21,612 unique children) from the test sample. The AUC scores for high-SES children are based on 9,462 referrals (representing 5,747 unique children) from the test sample. The estimation and evaluation period starts in April 2016 and ends in December 2017. Standard errors were calculated by boostrap using test sample data.

Table B.4. Results for children of different ages

	Limited set of covariates				Full set of covariates			
Method	AUC-ROC	95% CI		AUC-PR	AUC-ROC	95% CI		AUC-PR
**Panel A: Children aged 0–5**								
Logistic regression	83.61	81.26	85.96	14.34	85.13	82.93	87.33	18.20
Logistic regression w. LASSO	82.75	80.38	85.13	11.68	84.89	82.88	86.91	15.52
Random forest	85.58	83.17	87.99	23.19	88.62	86.46	90.77	36.08
XGBoost	86.66	84.37	88.96	31.21	89.82	87.92	91.72	38.45
Removal rate: 2.10%								
**Panel B: Children aged 6–14**								
Logistic regression	84.29	83.15	85.44	16.38	85.21	84.15	86.28	16.24
Logistic regression w. LASSO	84.02	82.89	85.14	15.62	85.50	84.43	86.56	17.26
Random forest	85.54	84.45	86.63	18.26	86.50	85.52	87.49	17.95
XGBoost	85.62	84.53	86.71	18.12	86.99	86.05	87.92	18.86
Placement rate: 3.16%								
**Panel C: Children aged 15–17**								
Logistic regression	79.27	77.49	81.04	21.07	79.91	78.13	81.69	18.59
Logistic regression w. LASSO	79.49	77.71	81.26	21.85	80.50	78.75	82.24	20.34
Random forest	82.21	80.58	83.83	22.24	82.39	80.78	84.01	20.45
XGBoost	81.57	79.92	83.21	19.41	82.24	80.67	83.81	19.74
Removal rate: 5.87%								

Notes: The table provides AUC scores and the associated 95% confidence interval for the four predictive models. The AUC scores for the 0–5-year-old children are based on 13,262 referrals (representing 7,135 unique children) from the test sample. The AUC scores for the 6–14-year-old children are based on 30,455 referrals (representing 16,229 unique children) from the test sample. The AUC scores for the 15–17-year-old children are based on 8,932 referrals (representing 4,947 unique children) from the test sample. The estimation and evaluation period starts in April 2016 and ends in December 2017. Standard errors were calculated by boostrap using test sample data.

C Additional external validation results

In this section, we provide additional evidence for the appropriateness of using child removal as an indicator of child maltreatment. To this end, we show in Table C.1 the prevalence of twelve additional adverse outcomes measured in 2017 for the three groups considered in Table 3: (i) the entire population of children residing in Denmark as of January 1, 2017 who were not involved in any referral during 2017; (ii) children involved in a referral in 2017 without a subsequent removal; and (iii) children involved in a referral in 2017 with a subsequent removal.

In Fig C.1, we show the average values of these outcomes measured in 2018 as a function of the risk scores generated by the XGBoost model. In doing so, we focus on the 30% of the sample that constitutes the test sample.

Table C.1. Indicators of maltreatment in three distinct groups

	(i)	(ii)	(iii)
Self-harm (0/1)	0.000	0.003	0.009
Diagnosed with ADHD (0/1)	0.008	0.055	0.106
Diagnosed with a conduct disorder (0/1)	0.000	0.008	0.027
Diagnosed with a personality disorder (0/1)	0.000	0.004	0.024
Diagnosed with a depression (0/1)	0.001	0.015	0.021
Diagnosed with an eating disorder (0/1)	0.001	0.006	0.011
Social well-being in school*	0.022	−0.329	−0.444
Neuroticism*	−0.028	0.430	0.574
Agreeableness*	0.022	−0.328	−0.542
Conscientiousness*	0.032	−0.483	−0.648
Academic self-perception*	0.026	−0.411	−0.473
Fraction of teeth with gingivitis	0.024	0.042	0.048
Number of individuals	1,107,088	65,131	3,609

Notes: The table shows the average value of a list of adverse outcomes measured in 2017 for (i) the entire population of children residing in Denmark as of January 1, 2017 who were not involved in any referral during 2017; (ii) children involved in a referral in 2017 without a subsequent removal; and (iii) children involved in a referral in 2017 with a subsequent removal. The variables marked with * indicate the use of standardized variables (z-scores).

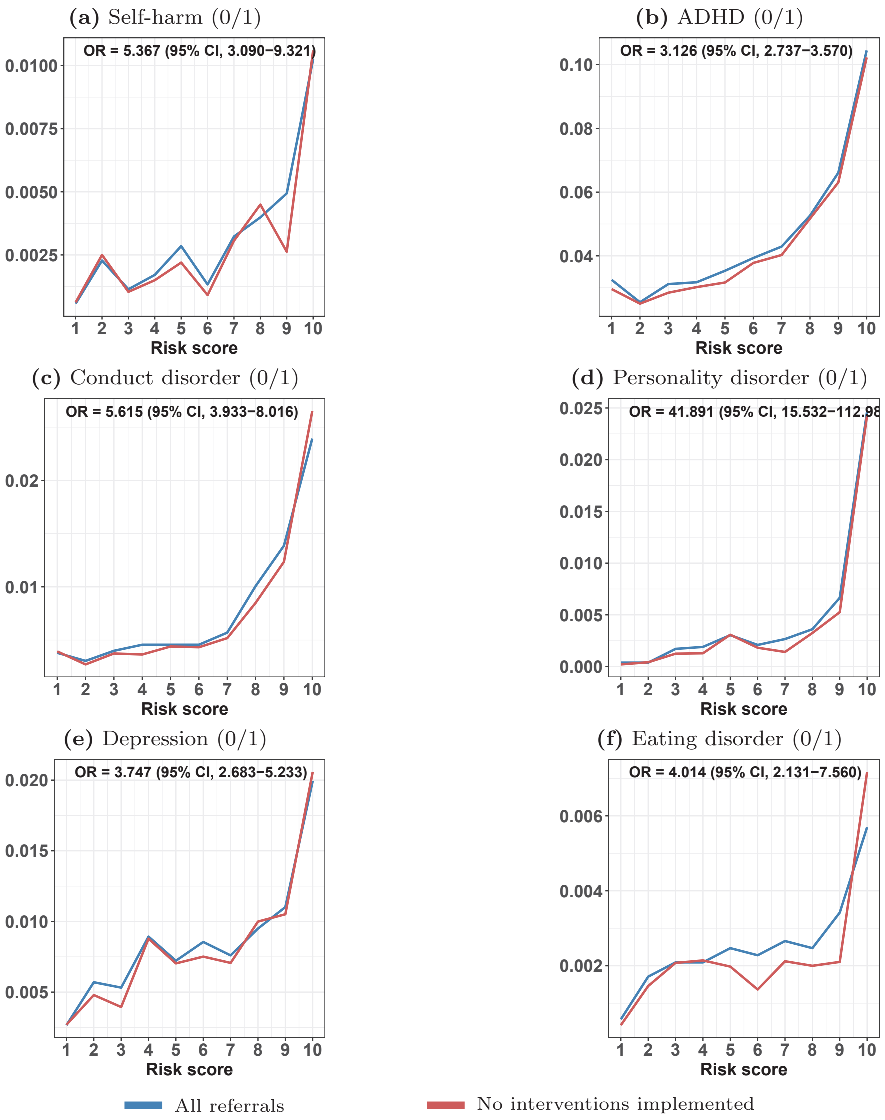

Fig C.1. This figure shows the relationship between the predictions of the XGBoost models with the full information set and the risk of adverse child outcomes. Only the individuals in the test sample are used for this analysis. The blue line represents all referrals in the test sample, whereas the red line corresponds to the set of referrals in which CPS implemented no interventions in the first four months. The odds ratios for the binary outcomes, compare the odds of the outcomes for risk scores 9 and 10 (high-risk cases) relative to the odds for risk scores 1 and 2 (low-risk cases) using all referrals. Figures (g)–(k) are calculated only for children of compulsory school age (6–16) and are standardized to have zero mean and unit variance at the population level.

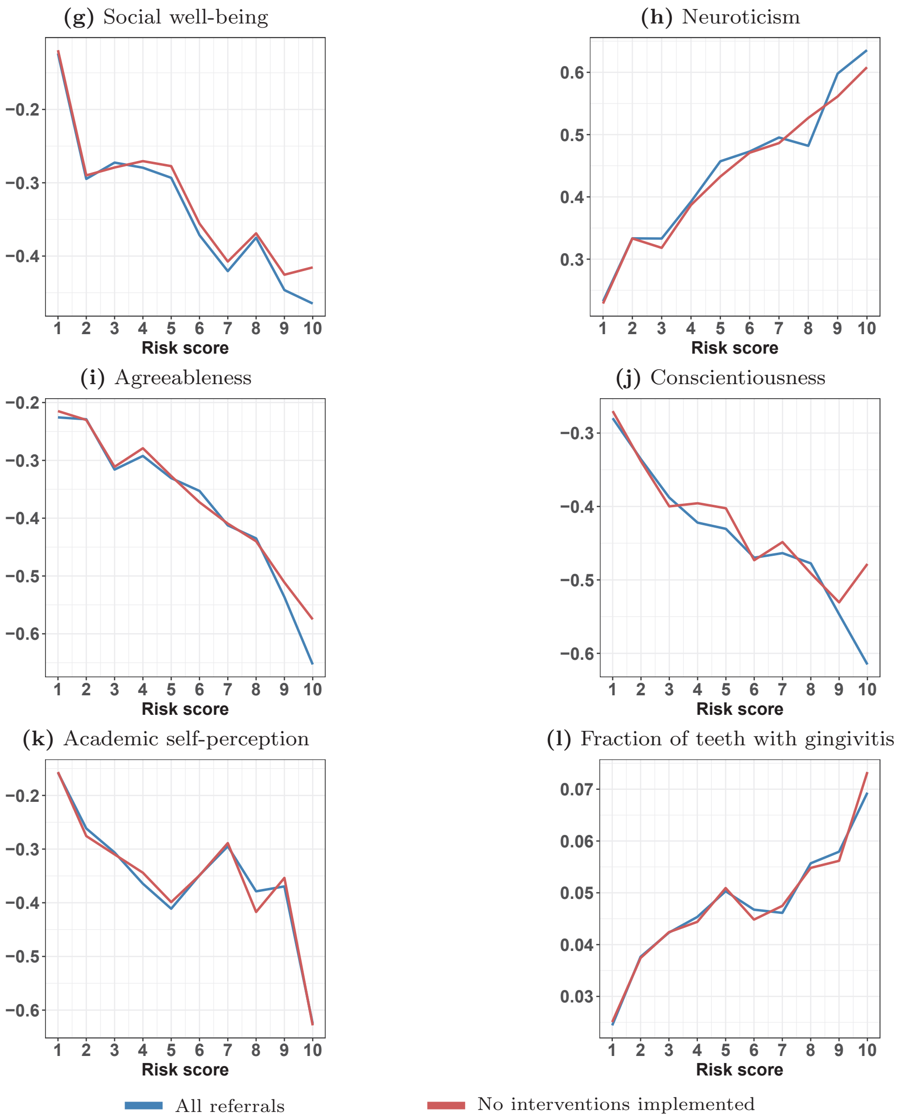

Fig C.1. (*Continued*) This figure shows the relationship between the predictions of the XGBoost models with the full information set and the risk of adverse child outcomes. Only the individuals in the test sample are used for this analysis. The blue line represents all referrals in the test sample, whereas the red line corresponds to the set of referrals in which CPS implemented no interventions in the first four months. The odds ratios for the binary outcomes, compare the odds of the outcomes for risk scores 9 and 10 (high-risk cases) relative to the odds for risk scores 1 and 2 (low-risk cases) using all referrals. Figures (g)–(k) are calculated only for children of compulsory school age (6–16) and are standardized to have zero mean and unit variance at the population level.

D Characteristics of misclassified referrals

Table D.1. Average characteristics of the individuals in the test sample

				CPS mistakes: Limited information set		CPS mistakes: Full information set	
	All cases	Removals	CPS mistakes	Risk score ≥ 9	Risk score < 9	Risk score ≥ 9	Risk score < 9
Age	9.762	11.515	10.796	12.382	10.085	12.362	10.083
Female (0/1)	0.458	0.467	0.464	0.466	0.464	0.461	0.466
Number of prior referrals	2.488	5.272	2.982	4.805	2.165	4.612	2.240
No prior referral (0/1)	0.338	0.065	0.222	0.037	0.305	0.044	0.303
Number of prior preventive services	0.273	0.755	0.415	0.923	0.188	0.926	0.183
Number of prior removals	0.032	0.236	0.046	0.148	0.001	0.148	0.000
Non-Western origin (0/1)	0.189	0.171	0.169	0.157	0.174	0.161	0.172
Low SES (0/1)	0.820	0.886	0.846	0.899	0.822	0.900	0.821
Charged (0/1)	0.241	0.452	0.297	0.409	0.179	0.403	0.182
Victimized (0/1)	0.057	0.184	0.070	0.120	0.048	0.147	0.035
Somatic illness (0/1)	0.379	0.443	0.394	0.435	0.375	0.443	0.371
Fracture (0/1)	0.152	0.201	0.174	0.210	0.158	0.218	0.154
Mental illness (0/1)	0.109	0.225	0.156	0.214	0.129	0.238	0.118
Anxiety (0/1)	0.049	0.129	0.074	0.109	0.059	0.120	0.053
Fraction of damaged teeth	0.069	0.061	0.070	0.072	0.069	0.074	0.068
Share of unauthorized school absence	0.031	0.051	0.038	0.057	0.029	0.059	0.028

Notes: In this table, we report the average characteristics of the individuals in the test sample with respect to their treatment status. The data is based on information in the year before the referral time. We define a CPS mistake as a referral for which no intervention (preventive service or removal) was initiated by CPS within the first four months, but where an intervention was initiated in the following eight months.

E Analysis results with age-corrected risk scores

In this appendix, we redo the parts of the analysis from the main text which depend upon the generated risk scores from the PRM. In the main text, the risk scores are generated by finding the deciles of the unconditional distribution of the predicted probabilities of out-of-home-placement within four months from the time of referral. However, as noted in the paper, child removals mainly occur for teenagers and newborns. This has the consequence that there will be a tendency for the high-risk scores to be overrepresented in these two subgroups. To avoid this pattern, we introduce in this appendix age-neutralized risk scores, which are generated by constructing decile risk scores based on the age-specific distribution of the predicted probabilities. By construction, this approach yields an equal distribution of risk scores across all ages.

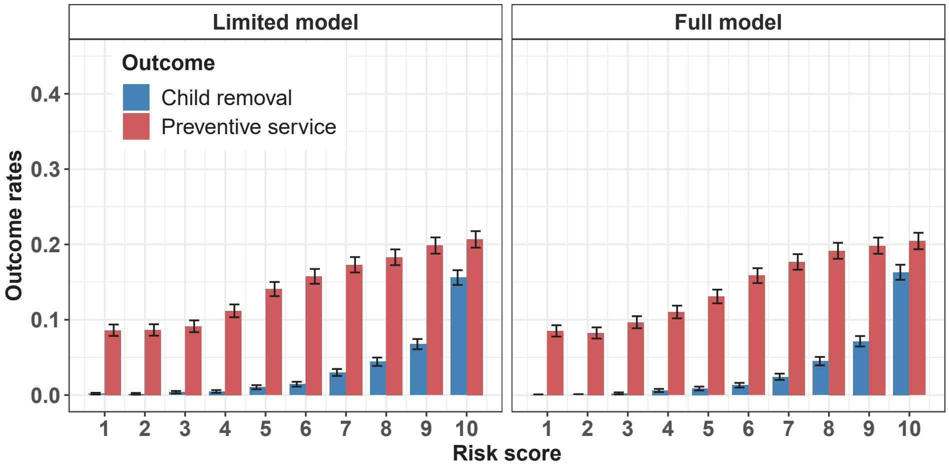

Fig E.1. The figure illustrates the observed rates of child removals and preventive services as a function of the age-corrected risk scores generated by the XGBoost model, during the four-month period following the receipt of a referral. The figure is only based on test sample data. The vertical error bars correspond to the 95% confidence intervals.

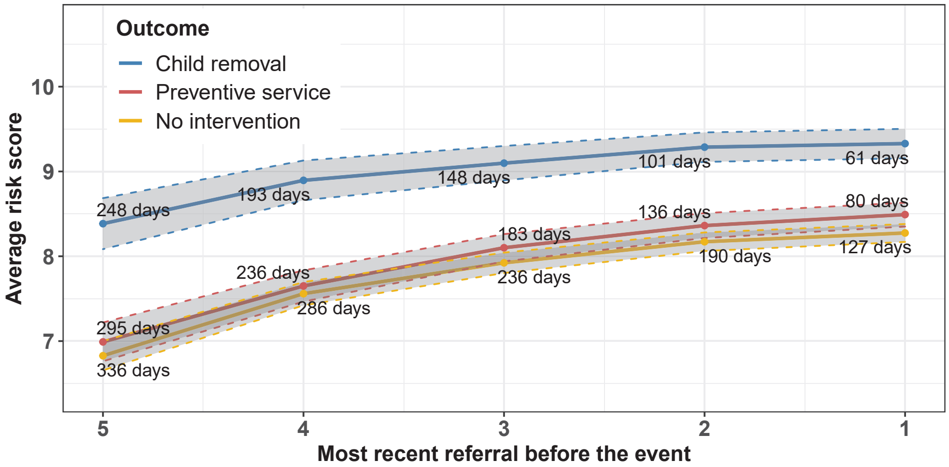

Fig E.2. This figure depicts the average age-corrected risk scores generated by the XGBoost model using full information for the most recent five referrals leading up to the initiation of a CPS intervention. The test sample is divided into three distinct subsets based on the status of the children from April 2017 to December 2017: (i) children who experience removal; (ii) children for whom a preventive service is initiated but no removal is made; and (iii) children for whom no intervention is implemented. For the latter group, we randomly assign a placebo intervention date between April 2017 and December 2017. The numbers displayed in the graph indicate the average number of days between the intervention date and the five most recent referrals.

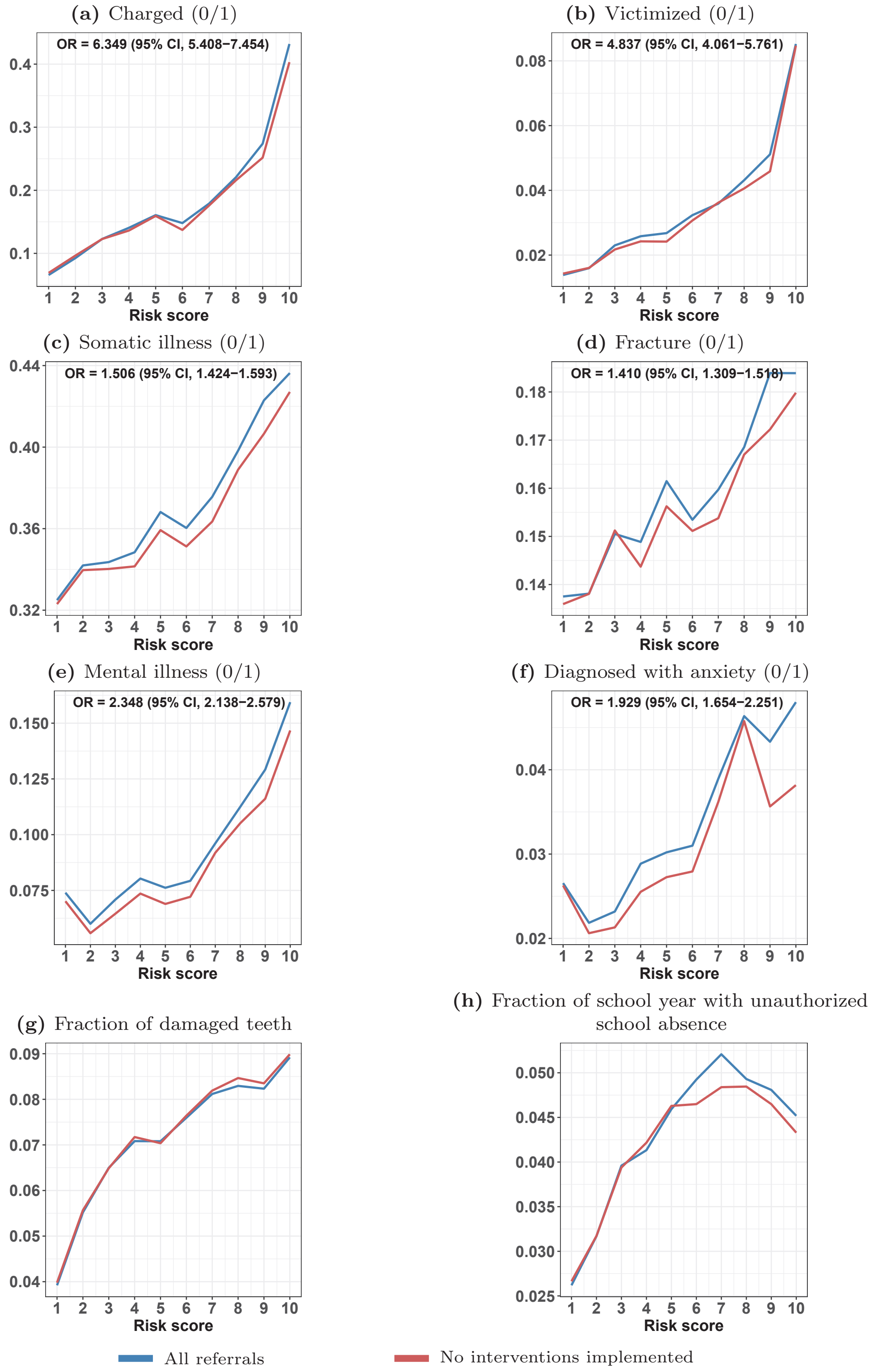

Fig E.3. This figure shows the relationship between the age-corrected risk scores of the XGBoost models with the full information set and the risk of adverse child outcomes. Only the individuals in the test sample are used for this analysis. The blue line represents all referrals in the test sample, whereas the red line corresponds to the set of referrals in which CPS implemented no interventions in the first four months. The odds ratios for the binary outcomes, compare the odds of the outcomes for risk scores 9 and 10 (high-risk cases) relative to the odds for risk scores 1 and 2 (low-risk cases) using all referrals. The numbers for charges are based only on children above the minimum age of criminal responsibility (15 years), while the numbers for school absence are based on children of compulsory school age (6–16 years).

Table E.1. Can predictive models help CPS identify cases of maltreatment?

	All referrals			No intervention within four months		
		Share of referrals with a risk score ≥ 9			Share of referrals with a risk score ≥ 9	
	Share	Limited information set	Full information	Share	Limited information set	Full information
**Preventive service:**						
(a) Within 4 months	0.153	0.308	0.306			
(b) Between 4 and 8 months	0.075	0.272	0.271	0.087	0.255	0.255
(c) Between 8 and 12 months	0.036	0.252	0.264	0.042	0.223	0.234
**Child removal:**						
(d) Within 4 months	0.034	0.668	0.701			
(e) Between 4 and 8 months	0.020	0.561	0.593	0.018	0.559	0.592
(f) Between 8 and 12 months	0.017	0.542	0.563	0.014	0.570	0.584
(a) or (d)	0.177	0.355	0.359			
(b) or (e)	0.084	0.298	0.303	0.102	0.298	0.303
(c) or (f)	0.041	0.279	0.289	0.050	0.279	0.289

Notes: The table shows the share of referrals in the test sample for which CPS initiated an intervention in response to a referral. The risk scores in this table are age-corrected and generated by the XGBoost model.

Table E.2. Average characteristics of the individuals in the test sample

				CPS mistakes: Limited information set		CPS mistakes: Full information set	
	All cases	Removals	CPS mistakes	Risk score ≥ 9	Risk score < 9	Risk score ≥ 9	Risk score < 9
Age	9.762	11.515	10.796	10.518	10.910	10.512	10.914
Female (0/1)	0.458	0.467	0.464	0.442	0.474	0.451	0.470
Number of prior referrals	2.488	5.272	2.982	4.982	2.158	4.852	2.202
No prior referral (0/1)	0.338	0.065	0.222	0.029	0.302	0.038	0.298
Number of prior preventive services	0.273	0.755	0.415	0.916	0.209	0.919	0.205
Number of prior removals	0.032	0.236	0.046	0.153	0.002	0.157	0.000
Non-Western origin (0/1)	0.189	0.171	0.169	0.161	0.172	0.172	0.167
Low SES (0/1)	0.820	0.886	0.846	0.913	0.818	0.924	0.813
Charged (0/1)	0.241	0.452	0.297	0.510	0.213	0.508	0.220
Victimized (0/1)	0.057	0.184	0.070	0.113	0.052	0.153	0.035
Somatic illness (0/1)	0.379	0.443	0.394	0.427	0.380	0.433	0.377
Fracture (0/1)	0.152	0.201	0.174	0.198	0.164	0.191	0.167
Mental illness (0/1)	0.109	0.225	0.156	0.185	0.143	0.204	0.135
Anxiety (0/1)	0.049	0.129	0.074	0.091	0.067	0.095	0.065
Fraction of damaged teeth	0.069	0.061	0.070	0.075	0.068	0.075	0.068
Share of unauthorized school absence	0.031	0.051	0.038	0.044	0.036	0.042	0.037

Notes: In this table, we report the average characteristics of the individuals in the test sample with respect to their treatment status. The data is based on information in the year before the referral time. The risk scores in this table have been age-corrected. We define a CPS mistake as a referral for which no intervention was initiated by CPS within the first four months, but where an intervention was initiated in the following eight months.

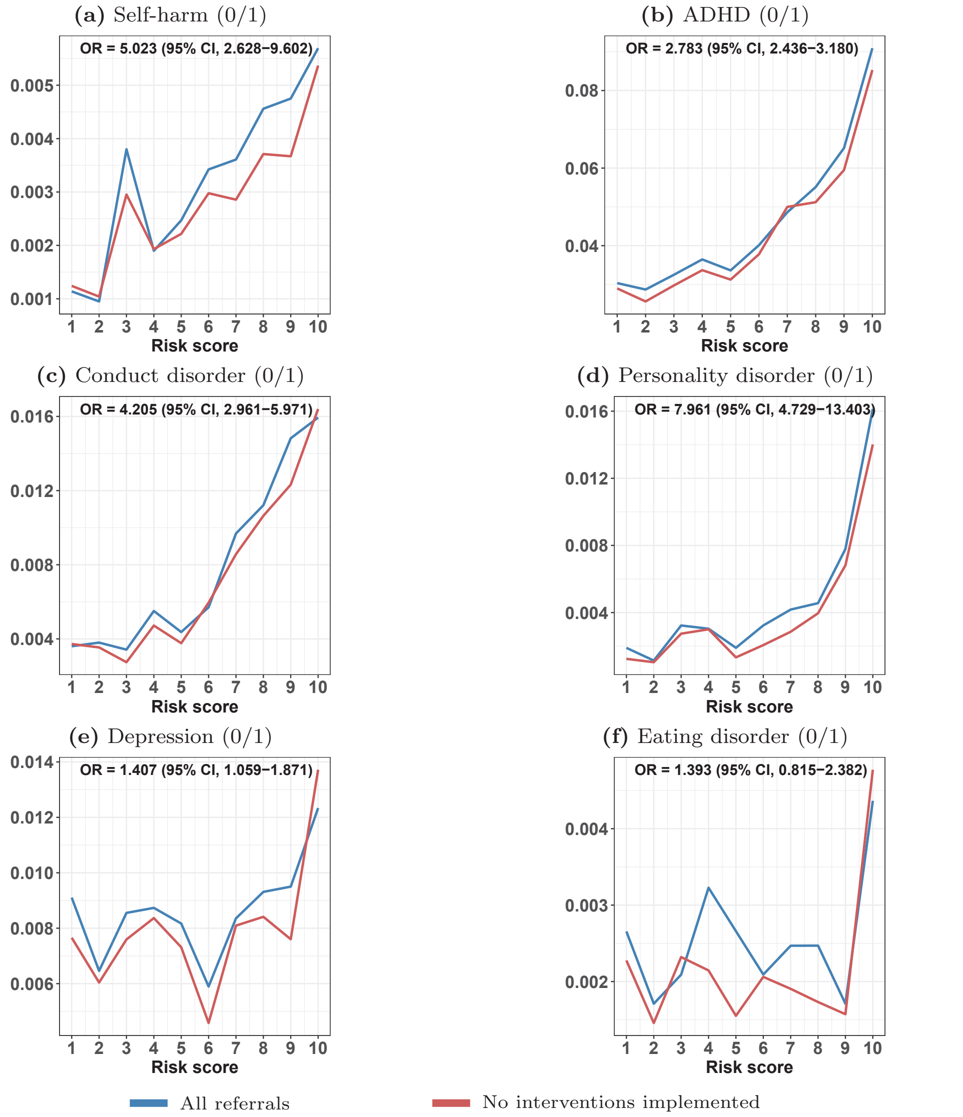

Fig E.4. This figure shows the relationship between the age-corrected risk scores of the XGBoost models with the full information set and the risk of adverse child outcomes. Only the individuals in the test sample are used for this analysis. The blue line represents all referrals in the test sample, whereas the red line corresponds to the set of referrals in which CPS implemented no interventions in the first four months. The odds ratios for the binary outcomes, compare the odds of the outcomes for risk scores 9 and 10 (high-risk cases) relative to the odds for risk scores 1 and 2 (low-risk cases) using all referrals. Figures (g)–(k) are calculated only for children of compulsory school age (6–16) and are standardized to have zero mean and unit variance at the population level.

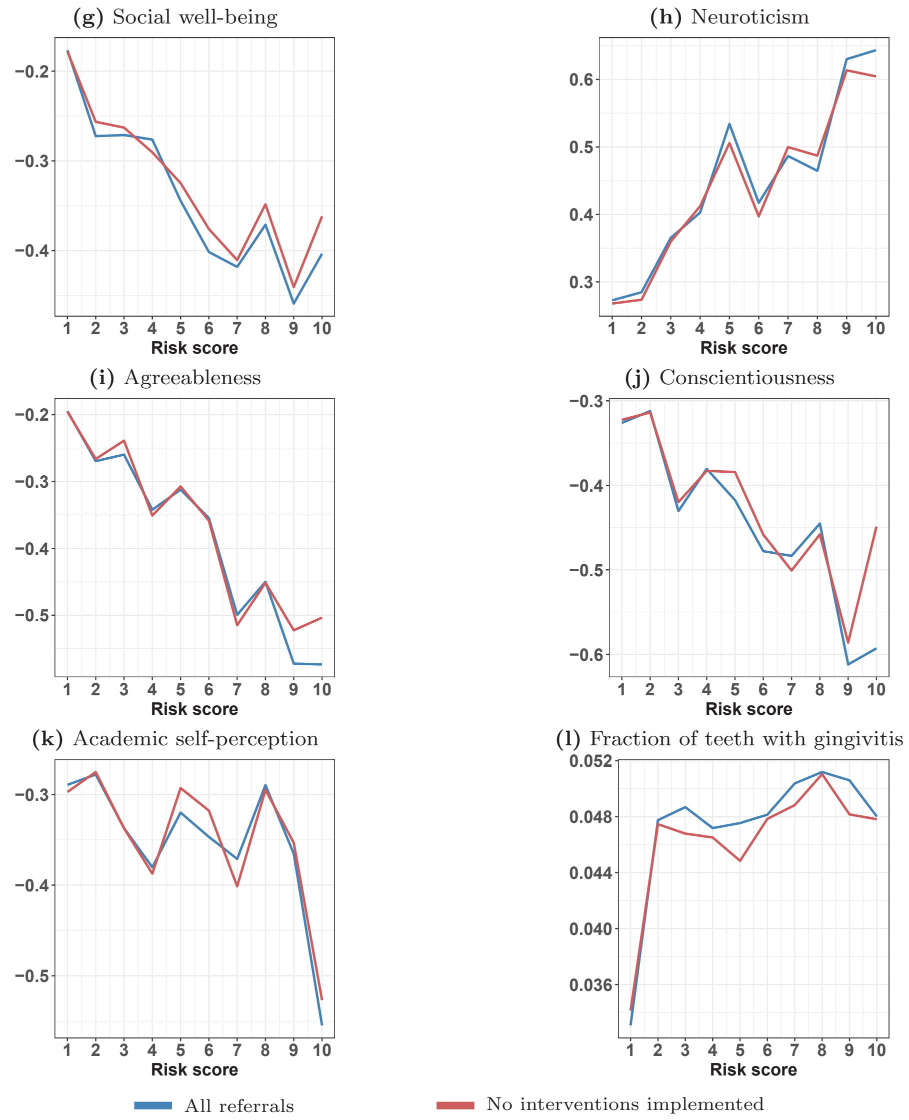

Fig E.4. (*Continued*) This figure shows the relationship between the age-corrected risk scores of the XGBoost models with the full information set and the risk of adverse child outcomes. Only the individuals in the test sample are used for this analysis. The blue line represents all referrals in the test sample, whereas the red line corresponds to the set of referrals in which CPS implemented no interventions in the first four months. The odds ratios for the binary outcomes, compare the odds of the outcomes for risk scores 9 and 10 (high-risk cases) relative to the odds for risk scores 1 and 2 (low-risk cases) using all referrals. Figures (g)–(k) are calculated only for children of compulsory school age (6–16) and are standardized to have zero mean and unit variance at the population level.
